# Automatic Design of Digital Synthetic Gene Circuits

**DOI:** 10.1371/journal.pcbi.1001083

**Published:** 2011-02-17

**Authors:** Mario A. Marchisio, Jörg Stelling

**Affiliations:** Department of Biosystems Science and Engineering and Swiss Institute of Bioinformatics, ETH Zurich, Basel, Switzerland; University of Virginia, United States of America

## Abstract

*De novo* computational design of synthetic gene circuits that achieve well-defined target functions is a hard task. Existing, brute-force approaches run optimization algorithms on the structure and on the kinetic parameter values of the network. However, more direct rational methods for automatic circuit design are lacking. Focusing on digital synthetic gene circuits, we developed a methodology and a corresponding tool for *in silico* automatic design. For a given truth table that specifies a circuit's input–output relations, our algorithm generates and ranks several possible circuit schemes without the need for any optimization. Logic behavior is reproduced by the action of regulatory factors and chemicals on the promoters and on the ribosome binding sites of biological Boolean gates. Simulations of circuits with up to four inputs show a faithful and unequivocal truth table representation, even under parametric perturbations and stochastic noise. A comparison with already implemented circuits, in addition, reveals the potential for simpler designs with the same function. Therefore, we expect the method to help both in devising new circuits and in simplifying existing solutions.

## Author Summary

Synthetic Biology is a novel discipline that aims at the construction of new biological systems able to perform specific tasks. Following the example of electrical engineering, most of the synthetic systems so far realized look like circuits where smaller DNA-encoded components are interconnected by the exchange of different kinds of molecules. According to this modular approach, we developed, in a previous work, a tool for the visual design of new genetic circuits whose components are displayed on the computer screen and connected through hypothetical wires where molecules flow. Here, we present an extension of this tool that automatically computes the structure of a digital gene circuit–where the inputs and the output take only 0/1 values–by applying procedures commonly used in electrical engineering to biology. In this way, our method generalizes and simplifies the design of genetic circuits far more complex than the ones so far realized. Moreover, different from other currently used methods, our approach limits the use of optimization procedures and drastically reduces the computational time necessary to derive the circuit structure. Future improvements can be achieved by exploiting some more biological mechanisms able to mimic Boolean behavior, without a substantial growth of the algorithmic complexity.

## Introduction

A central concept of Synthetic Biology [1] is the rational design of synthetic gene circuits by means of modularized, standard parts, which are DNA traits with well-defined functions. The field aims at adapting methods and ideas–such as part *composability* and *abstraction hierarchy*–from (electrical) engineering to biology. Several computational tools embracing these concepts have been developed (see [2] for a review). Moreover, some tools permit to realize circuits in a *drag and drop* way as it is typical in electronics [3]–[5]. Nevertheless, *de novo* design of circuits able to reproduce a target function is not an easy task and its *automation* represents a major challenge in Synthetic Biology.

Previously, François and Hakim [6] showed that small networks characterized by a desired behavior can be obtained by evolutionary optimization of a set of independent circuits. Similar optimization-based tools like Genetdes [7] and OptCircuit [8] use simulated annealing and mixed integer dynamic optimization, respectively. These approaches yielded interesting circuit designs, but they have several inherent limitations. Computational complexity requires very simplified models that do not represent basic parts but lump functionalities of entire genes. Similarly, brute-force optimization can only cope with rather small networks, and it requires dual optimization of circuit structure and of kinetic parameter values. Hence, more direct, rational design methods are desired.

Here, instead of looking for a general solution to the automatic design challenge, we focus on *digital* (logical) circuits. These circuits employ Boolean (binary) logic where input and output signals can take only two values: 0 (low signal) and 1 (high signal). In the simplest case, a Boolean *gate* uses two input signals to compute a single logical output. More complex digital circuits convert 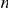 inputs into a single output. In both cases, the input-output relation is represented by a *truth table* where each entry specifies one of the possible 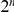 combinations of input signal values and the corresponding binary output.

In biology, digital circuits are important for several reasons. First, logical gates such as those determined by the action of two different activators on a promoter are abundant in natural systems. They are often found in association with feed-forward loop (FFL) motifs and provide more complicated functionalities such as sign sensitive delays [9], [10] and pulse generation [11]. More complex networks of several FFLs interacting with basic Boolean gates control sporulation in *B. subtilis* [12] as well as the neuronal system of *C. elegans* [13] (see [14] for a recent review). An analysis of possible implementations of logical gates could, thus, help further our understanding of natural biological networks.

In synthetic biology, secondly, complex digital circuits are required for the construction of biosensors and molecular computers. Biosensors should respond to well-defined external cues that may be specified with a truth table. The more inputs can be sensed, the better is the ability of the (digital) biosensor to discriminate between similar environmental conditions. Such biosensors could be integrated, for instance, into bioreactors for the production of biofuels [15]. Furthermore, they could play an important role in disease treatment—Anderson *et al.* [16] implemented a biosensor that mimics a logical gate to control bacterial invasion of tumor cells in response to signals from the tumor environment. Even more complex biosensors could work as molecular computers that perform a diagnosis on the basis of the sensed substances and release drugs if necessary [17].

Motivated by these two aspects, several synthetic gene circuits that implement Boolean logic have been realized experimentally in the past years (e.g. [18]–[23]). Most of these circuits rely on transcriptional control schemes. In fact, it is well known that bacterial promoters can display logic behavior when controlled by two transcription factors [24]–[26]. More complex “Boolean” promoters have been engineered, for instance, in mammalian cells [27]. However, the number of repressors and activators generally used in synthetic biology is low and the rational engineering of transcription factors can be a complex process [28].

Alternatively, Boolean gates are achieved in nature by mechanisms of translation control like base-pairing between antisense small-RNAs and the mRNA, or structural mRNA modifications due to the binding of chemical effectors (e.g. thiamine and tetracycline) to riboswitches and ribozymes [29], [30]. These are complex RNA structures made of two modules: an *aptamer*, where a chemical binds, and an *actuator* that either undergoes structural modifications in a riboswitch or gets spliced in a ribozyme as a consequence of the chemical binding. Both riboswitches and ribozymes can either repress or activate translation [30]. Furthermore, a *tandem* riboswitch, where a single actuator is under the control of two aptamers, has been observed in *B. clausii* [31]. With two distinct inputs, it represents a natural Boolean gate located on the mRNA. Taking these structures as models, similar synthetic RNA constructs have been engineered recently [32]. In particular, Win and Smolke [33] have built complex ribozymes that establish the most common two-input Boolean gates. Importantly, the design of small RNAs is easy compared to the design of transcription factors.

Despite these individual successes, synthetic biology fundamentally lacks tools and concepts for automatic computational design. Logical circuits are suitable starting points for automatic design because the target function can be defined easily by a *truth table*. Here, we combine approaches from electrical circuit design with our previous model for circuit design with composable parts [4], [34] to develop a method for the *automatic design* of digital synthetic gene circuits. It is implemented as an add-on for the process modeling tool ProMoT [35], [36]. The circuits use a set of standard biological parts and Boolean gates whose kinetic parameters take appropriate default values without invoking any optimization algorithms. In addition to previously developed building blocks such as two-operator-containing promoters, we consider externally controllable ribosome binding sites (RBSs). The method requires only a truth table to directly produce several possible circuit designs that process up to four different inputs to yield a unique, pre-defined output signal. Design alternatives are ranked according to a *complexity score* that reflects the efforts for practical implementation. Simulations on single gates and on networks of different complexity confirm the validity of our approach by highlighting accurate representation of the truth table and robustness of the designed circuits.

## Results

### A set of biological Boolean gates

**Figure 1:**
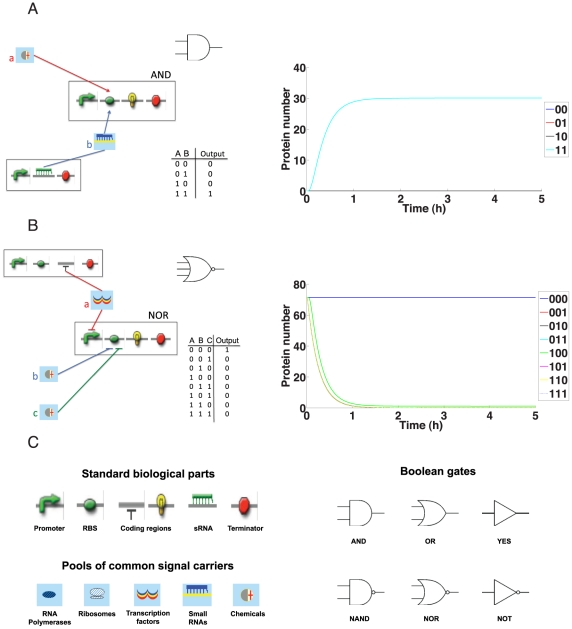
Biological Boolean gates. The simple composition of standard biological parts permits to build Boolean gates with different numbers of inputs. (A) Configuration of a two-input AND gate. A constitutive promoter is flanked by an RBS with two hairpins, one of which coincides with a riboswitch. These mRNA structures prevent ribosomes from binding the RBS and represent the targets of two different inputs: a chemical, which binds the riboswitch, and a small RNA, which is complementary to the other hairpin. Only both inputs together remove all structural hurdles and allow translation initiation. This confers an overall AND logic function to the construct. (B) Configuration of a three-input NOR gate. The RBS contains a tandem riboswitch that, in its ground state, does not form an obstacle for ribosome binding. However, when at least one input signal (chemical) reaches the corresponding aptamer, the riboswitch changes its configuration and closes the access to the RBS. Additionally, the promoter is controlled by a repressor. Hence, RNA polymerase can start transcription only when no negative transcription factor is synthesized. Overall, this gene produces a reporter protein only when all the three inputs are absent. Thus, it performs a NOR logic operation. (C) Formal symbols of standard biological parts, pools, and Boolean gates employed throughout.

In electrical engineering, digital circuits are organized into Boolean *gates* that establish basic logical operations. AND gates perform a multiplication of the input signals: they return a 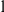 only when all inputs are high (Fig. 1A; see also Fig. 1C for a definition of symbols). Similarly, a NOT gate converts a low signal into a high one or vice-versa and YES gates produce a logical output that is identical to the input. OR gates compute the sum of (at least) two inputs; they give a 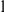 output when at least one input is high. Complementary operations are performed by *negated gates*. For a NOR gate, a 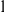 results only if all inputs are low (Fig. 1B), and in a NAND gate, in contrast, a 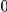 is produced when all inputs are high. Other gates achieve more complex operations. An XOR (exclusive OR) gate, for instance, returns a 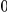 only when all inputs are either high or low, and a 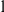 otherwise (see Text S1 for details).

Here, we employ standard biological parts (Fig. 1C), namely promoters, ribosome binding sites, small RNA (sRNA), protein (transcription factor and reporter) coding regions, and terminators for the design of corresponding logical circuits. Biological Boolean gates arise from the composition of standard parts into one or more transcription units. However, logic behavior is due only to particular configurations of promoters and RBSs. For instance, the AND gate shown in Fig. 1A is composed of one transcriptional unit with two inputs, namely a chemical (a) and an sRNA (b) produced by a second transcriptional unit. It is the particular configuration of the RBS, where either input can activate translation of the reporter protein, that generates the desired logical behavior.

More generally, computational approaches for the rational design of biological Boolean gates, so far, employed either only activation and repression of promoters [7], [8], or exhibited complex structures embracing more than one single transcription unit (see [37] for an example). Translational regulation processes–although experimentally exploited [38]–have been substantially neglected. The two example gates shown in Fig. 1A–B, in contrast, rely on our new design for Boolean gates that combines transcriptional and translational control. Promoters are regulated by the binding of transcription factors that, in turn, are activated by chemicals. In addition, we developed mathematical models for RBSs where basic Booleans gates with one or two inputs are achieved either through the binding of chemicals to single and tandem riboswitches or via mRNA base pairing with up to two sRNAs that act as “repressors” (*locks*) or as “activators” (*keys*) [39]. Following [40], cooperativity between the chemical receptors (*aptamers*) of tandem riboswitches is reproduced and, as a novelty, mixed configurations, where one sRNA binding site is accompanied by a riboswitch, are allowed. For example, the NOR gate in Fig. 1B employs the coupled action of a repressor that acts on the promoter, and of two chemicals that target the RBS as in a tandem riboswitch. In our model based on full mass-action kinetics (see Text S1), we considered only single and tandem riboswitches [41], [42] and neglected ribozymes or more complex synthetic constructs [43], [44]. Note that sRNAs binding sites and riboswitches could also lie in the coding region but–without loss of generality–we neglected this possibility in our model.

Within this framework, one-input NOT and YES and two-input NOR and AND gates can be realized by single-input/dual-input promoters respectively, or by appropriately designed RBSs (see Materials and Methods). Alternatively, when two inputs are present, one can be sent to the promoter and the other to the RBS. These new configurations, when the corresponding models were parametrized with literature and biologically plausible parameter values, work well in terms of reproducing the desired truth table in dynamic simulations *in silico* (see Fig. 1A,B and Text S1). However, they still require experimental confirmation.

To engineer gates with more than two inputs, we have to consider that promoter and RBS of the same transcription unit are logically connected by an AND operation. Hence, three-input NOR/AND gates can be implemented just by connecting a two input NOR/AND gate on the promoter with an inducible/repressible RBS or vice versa. Fig. 1B shows an example for such a NOR gate; despite its higher complexity with three inputs, the simulation results demonstrate the desired logical circuit behavior. Four-input NOR/AND gates are given by joining a promoter and an RBS both implementing a two-input NOR/AND gate. Thus, with our model based on composable parts, we can construct Boolean gates–and consequently digital circuits–regulated by up to four inputs.

### An electronics-based approach to circuit design

**Figure 2:**
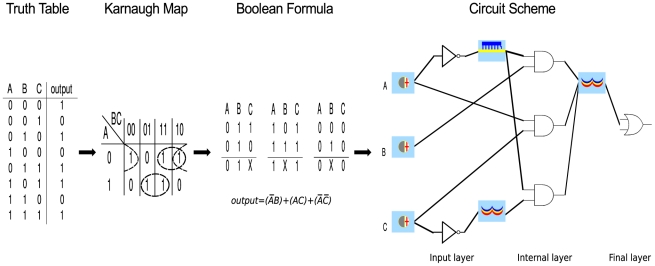
Conversion of a truth table into a circuit scheme via the Karnaugh map method. A Karnaugh map can be considered as a particular rearrangement of a truth table. Here, three Boolean variables (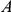, 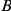 and 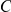) are taken into account. The values of 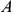 are written on the rows of the Karnaugh map, whereas the values of 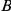 and 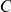 lie on its columns. The Karnaugh map method permits to derive both the SOP and POS form of the Boolean expression associated with any truth table. Here, only the SOP calculation is shown (see Text S1 for a more detailed explanation of the method). The circuit scheme follows straightforwardly: each variable that is negated in one or more clauses (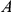 and 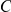 in the example) demands a NOT gate in the input layer. Every clause corresponds to an AND gate of the internal layer. An OR gate in the final layer gathers and sums the binary outputs of the internal AND gates. In the example, chemicals, sRNAs and transcription factors regulate the three AND gates that produce a unique kind of activator able to control the final OR gate.

In electronic circuit design, the construction of larger-scale digital circuits from well-defined Boolean gates is a standard procedure, and several computational methods for this task have been developed. One of these is called the ‘Karnaugh map method’ [45], [46], which we employ here for the automatic design of digital biological circuits. The algorithm starts with a truth table as the input (see Fig. 2 for an example circuit). By re-grouping (mapping) input-output relations of the specification, it allows a conversion into a Boolean formula that is usually composed of several logical terms. More specifically, the method permits to derive two different descriptions of every digital circuit that, in electronics, are called POS (Product Of Sums) and SOP (Sum Of Products, as in the example in Fig. 2) forms. An exclusive OR (XOR) gate, for instance, is given in POS as 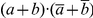 whereas in SOP as 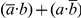, where 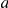 and 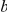 are the two input signals and 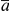 and 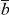 are their negations.

Both classes of Boolean formulas completely specify a circuit structure in terms of Boolean gates. Such a circuit is organized in three *layers*. The first layer contains a NOT gate for each negated input signal. A second layer employs as many OR/AND gates as the number of clauses in the corresponding formula–there are three clauses in our example of Fig. 2. The final layer uses a single AND/OR gate to gather the outputs of the gates in the previous layer. Depending on the type of gates in the second and third layer, the circuit logic is called either OR-AND (POS) or AND-OR (SOP). With *De Morgan's laws* [46], these forms can be converted into NOR-NOR and NAND-NAND logic, respectively, such that only one kind of gate is required besides the NOT in the input layer. A *minimal* formula involves the lowest number of gates. For the XOR gate, for instance, POS and SOP solutions are equivalent and require five gates each (see Fig. S1).

As in electronics, our digital genetic circuits are organized in three layers (Fig. 2). The POS representation employs the reduced NOR-NOR logic whereas for SOP only the non-reduced AND-OR logic is available. NAND gates required for SOP are not generally applicable since they cannot be built on sRNA regulation mechanisms (see Materials and Methods). The inputs for our designed digital circuits are chemicals. We define *inducers* as chemicals that activate an activator protein or inhibit a riboswitch that, in its unbound state, prevents ribosome binding. This kind of small molecules promote either transcription or translation, which is necessary for the construction of both OR and AND gates (see Materials and Methods). Hence, inducers are the input molecules for circuits in the SOP form. In addition, we call *corepressors* those chemicals that activate a repressor protein or provoke a structural change in a riboswitch such that it will prevent ribosome binding. Corepressors (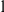 logic input) set protein or sRNA synthesis to minimal values (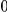 logic output). For this reason, they are the natural input to NOR gates and, as a consequence, to circuits in the POS form. Overall, both types of input chemicals act directly on a RBS or indirectly (after binding a transcription factor) on a promoter.

In contrast to electronics, the input layer of a synthetic circuit contains YES gates in addition to NOT gates. A YES gate is a simple transcription unit that synthesizes a transcription factor when an input chemical is present. On the contrary, NOT gates can show complex configurations–made of up to three transcription units–that are required to convert an input chemical into either a protein or a small RNA (see Fig. S2).

Chemicals can act directly on the gates of the second (*internal*) layer because they are valid inputs for properly designed riboswitches. In POS, internal gates are either NOR or NOT; in SOP, AND or YES. All these gates are single transcription units regulated by up to four inputs. The easiest way of implementing the third (*final*) layer is through a single gate. It corresponds to the operation of sum (OR, in SOP) or multiplication (NOR, in POS) that connects the clauses of the Boolean formula. The logic resides in a one-operator promoter or, less preferably, in a RBS regulated by a sRNA. All internal gates produce the same regulatory factor: an activator (or a key) in SOP to reflect the OR logic as in Fig. 2, and a repressor (or a lock) for the NOR logic in POS.

This *single* final gate solution, which is the direct translation of a Boolean formula into a circuit scheme, is clearly minimal in terms of gene and regulatory factor number. However, we explored other possible classes of final layer schemes, termed *disjoint* solutions (see Text S1 for details), in order to see if structurally more complex solutions can lead to a better circuit performance. Notice that these alternative solutions do not have a direct counterpart in electronics and do not require changes in the Karnaugh map algorithm. They are constructed by modifying the final layer of the single (basic) scheme.

Following the above considerations, we developed an algorithm for automatic circuits design that involves the following basic steps: (i) reading a pre-defined truth table, (ii) converting it into a Karnaugh map, (iii) deriving both POS and SOP circuit expressions, and (iv) implementing a selected solution using standard biological parts and gates (see Text S1 for details). Note that as a consequence of the electronics-like approach, only parameter values for the gates potentially need optimization, but not the circuit structure. Our current implementation allows the construction of digital circuits with up to four different inputs. Hundreds of possible circuit schemes for a given truth table are computed in just a few seconds (see Text S1); we will discuss computational limitations of this approach below.

### Circuit design alternatives and complexity

**Figure 3:**
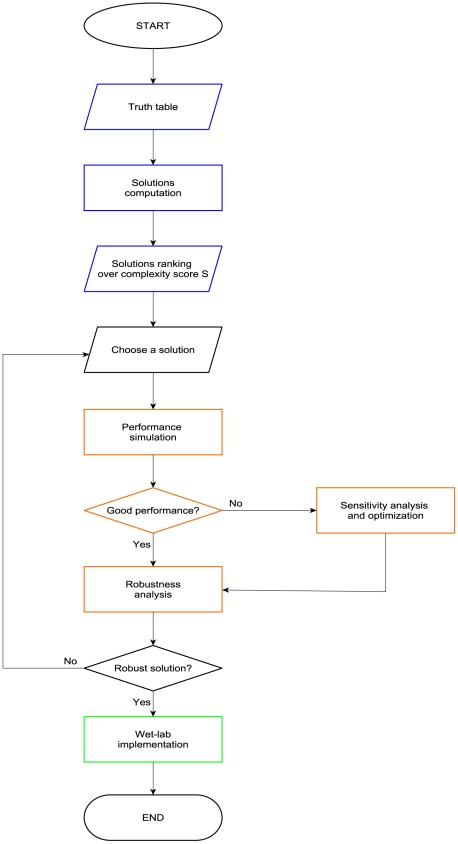
Workflow for digital gene circuit design. The overall procedure of constructing a digital, synthetic, gene circuit starts with the network automatic design that uses our computational tool based on the Karnaugh map method (blue boxes). A solution, normally the least complex one, undergoes other simulations to check and, if necessary, improve its performance and robustness (orange boxes). Finally, if the (optimized) solution meets the necessary requisites for a faithful reproduction of the corresponding truth table, it is implemented in the lab, otherwise another circuit solution has to be taken into account.

The complexity of electronic circuits essentially depends only on the number of gates—which can be minimized through the Karnaugh map method. Synthetic biology, however, is distinct from electronics in two important aspects. Firstly, circuit designs for a given truth table differ both in gene number, and in the kind and the quantity of regulatory factors. Both factors affect the possibilities of achieving a practical implementation of a design. Secondly, circuit performance and robustness are not guaranteed in biology due to nonlinear interactions within a designed circuit, and as a consequence of the circuit's embedding into a host cell. Ideally, complexity and performance/robustness would be considered simultaneously in identifying the best circuit design, but performance-based evaluations require large numbers of computationally expensive circuit simulations, which quickly becomes prohibitive. Therefore, we propose the workflow for digital circuit design shown in Fig. 3. Initially, it filters solutions based on a complexity score that reflects the main constraints for a wet-lab implementation. Then, only the chosen solution(s) undergoes more elaborate and time-consuming analysis—and potentially detailed optimization—steps to establish reliable performance *in vivo*.

To derive a complexity score, it is important to consider that each gate must be controlled by unique signal carriers such as transcription factors and sRNAs to avoid cross-talk between standard parts. For instance, a repressor binding both to an internal and to the final NOR gate would make the network fail to reproduce some truth table entries. Therefore, we estimate the *complexity* of a circuit solution as a function of the number of the regulatory factors involved in the network. Moreover, transcription factors and sRNAs determine circuit complexity in different ways. Beyond the few transcription factors often used in synthetic biology, such as the LacI, TetR, CI repressors, and the CRP activator, engineering new efficient transcriptional regulators is generally time-consuming and difficult. In contrast, sRNAs can be synthesized more easily, such that regulation via locks and keys should be employed whenever possible.

With this rationale, we introduce the score:
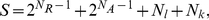
(1)to characterize a circuit solution's implementation complexity, where 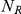, 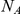, 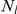, and 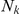 represent the total number of repressors, activators, locks and keys present in the circuit, respectively. The circuit complexity increases dramatically–following a power law–when the same type of transcription factor is used repeatedly, whereas the sRNA number weighs much less. Furthermore, solutions that are not particularly complex can exist despite a rather high number of regulatory factors, provided they are fairly equally distributed among the four available species (with a preference for the use of locks and keys). Note that for 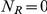 or 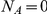, the corresponding term is removed from Eq. (1). We want to stress that 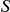 does not reflect the *quality* of a circuit design–the score is intended to characterize its practical realizability.

**Figure 4:**
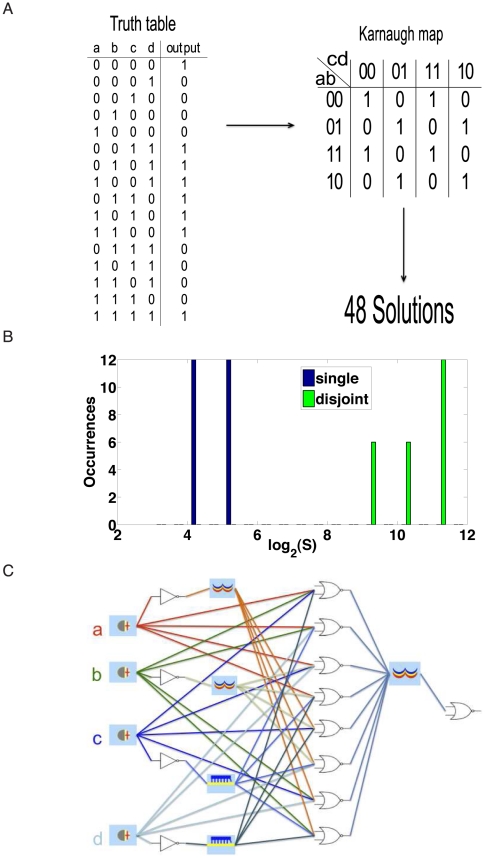
Example circuit (test case A). Test case A corresponds to the most complex Boolean formulas generated by our tool. (A) Truth table and Karnaugh map. (B) Solution distribution according to the complexity score. (C) Solution 1 scheme–the least complex one for test case A.

To test the validity of this score we considered a complex four-input circuit (Fig. 4A). We refer to this circuit as test case A (see Text S1 for more examples). Altogether, we obtained 48 circuit designs that are compatible with the specifications in principle. The distribution of complexity scores shows that single-gate structures are substantially less complex than the disjoint schemes (see Fig. 4B). Without such a score, evaluating the complexity of a circuit solution is not straightforward. For instance, Fig. 4C shows the least complex solution for the test case—for complicated structures such as this one, a quantification is required.

**Figure 5:**
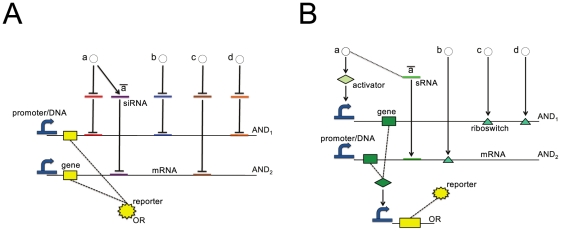
Comparison of a RNAi-based with an automatically designed circuit. The Boolean formula 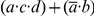 is here represented both (A) as the circuit provided by Rinaudo *et al.* [23] with 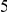 different siRNAs and (B) as one of the 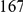 solutions computed by our tool, using two activators and one sRNA. Notice that 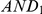 and 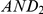 correspond to 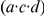 and 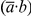, respectively. Dashed lines indicate either protein synthesis or input signal conversion into a regulatory factor (NOT operation). For a better comparison with Rinaudo's scheme, we do not include the input layer in (B).

Next, we were interested in comparing our design alternatives with existing natural or synthetic circuits that perform identical logical signal processing. For natural circuits such as bacterial transcriptional networks, the functions in terms of a truth table are usually not sufficiently characterized to enable such a comparison. However, recently Rinaudo *et al.* [23] constructed Boolean gates based on RNA interference exclusively and showed their functional operation in mammalian cells. One implemented example circuit is shown in Fig. 5A. Note that siRNA production, which is supposed to be either activated or repressed by endogenous signals that act as circuit inputs, is not shown in the gate design explicitly. The exact number of genes required to engineer this circuit is not known, but since 5 different siRNAs were necessary for its implementation, we can attribute a complexity score 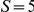 to the configuration.

With our method, we re-designed the circuit, that is, we determined all possible circuit solutions that comply with the truth table. In less than one second, our tool generated 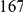 possible schemes. The least complex circuit (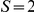) is in POS and the “best” solution in SOP has a complexity score 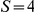. Overall, we found 15 configurations with a complexity score lower than five that require only between 5 and 7 genes (including the input layer). One example circuit is shown in Fig. 5B—the more compact implementation of the logic functions is directly evident from comparison with Fig. 5A. Importantly, this circuit complexity is not out of reach for *in vivo* implementation and, according to our simulations (see Text S1) our solution would reproduce the experimental results in [23]. Hence, our tool can provide valid, alternative designs to the ones so far adopted to build digital gene circuits in the wet lab (see Text S1 for details). By differentiating between design alternatives, the score is essential to judge potential implementability of a designed circuit.

### Circuit performance

To better understand the general relations between complexity and performance, we collected and simulated solutions in POS and SOP for test case A covering all the possible final layer structures.

**Figure 6:**
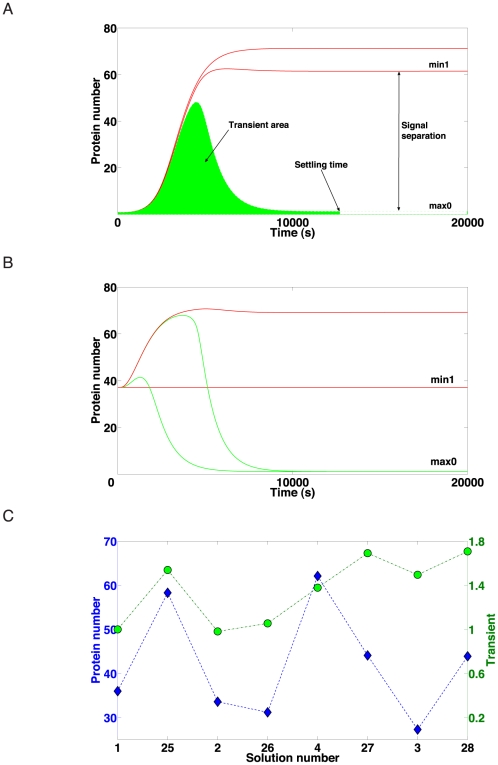
Circuit performance. (A) The parameters used to estimate the quality of a digital circuit are reported on the plot of a generic solution (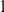 outputs lie between the two red lines, 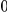 outputs on the green surface). (B) Test case A solution 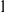 simulation. Only the results of four (out of sixteen) truth table entries are shown. All the 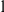 outputs lie between the red lines and all the 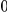 outputs between the green ones. Every simulation consisted of two steps. First, the system reached a first steady state in the absence of chemicals (not shown). Afterwards, input signals were sent to the circuit. As a response, the network varied the reporter protein production and settled to a new steady state that describes the output (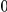 or 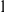) of the corresponding entry in the truth table. (C) Signal separation and transient for eight different solution of test case A. Transients have been rescaled with respect to the solution 1 value.

Mainly two factors determine the performance of a logical circuit, beyond reproducing the truth table in principle: the extent to which high and low output signals can be practically distinguished, and the transient dynamics after changes in the inputs that may–for a certain time–give incorrect results (see Fig. 6A for an illustration of typical circuit dynamics). To address the first issue, we computed the steady state *signal separation* (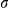). More specifically, we calculated the absolute signal separation as the difference between the minimal steady-state output for a logical 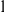 (*min1*) and the maximal 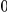 output (*max0*) (see Fig. 6A). Additionally, we considered the *ratio* (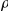) between 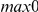 and 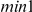 to separate between good and bad solutions (see Text S1).

Transient dynamics in circuit states may be critical for practical performance. In switching between states (or when the circuit is initialized), high and low outputs are indistinguishable and several peaks of different heights can be generated before reaching the final plateau. When a circuit controls other processes, the height and the duration of these peeks–mainly if associated with 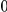 outputs–may cause undesired repercussions. We defined the solution *transient* as the average area covered by 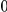 outputs during the time the circuit takes to get to the steady state (settling time, see Fig. 6A).

With these metrics, we comprehensively quantified the design alternatives' performance as summarized in Fig. 6C for test case A. Note that for the corresponding simulations, promoter and RBS basal production rate were set to very low values (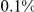 of the internal gate transcription/translation rate). An example for simulation results for the least complex circuit design (solution 1) is shown in Fig. 6B (see Text S1 for the other solutions). After an initial transient, the 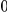 and 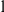 signals are clearly separated indicating that the circuit operates as specified by the truth table. The settling time is on the order of a few bacterial generation times, as observed for other synthetic circuits. In general, for the 8 solutions investigated in detail, the signal separation of 40–60 protein molecules (Fig. 6C) appears sufficient for practical detection in a biological cell. Not unexpectedly, we find a certain correlation between signal reliability and potentially harmful transients (Fig. 6C) with increasing circuit complexity. Overall, the simulations indicate that, for our Boolean gate design and parameter value choices, all circuits perform the intended function, but higher complexity may yield better performance. In any case, current technical limitations let us focus on the less complex *single* class circuit schemes in order to find circuit designs suitable for practical implementation.

In evaluating the performance of automatically designed circuits we employed standard parameter settings for all parts of the models, in particular, for the basic logical gates. Apparently, further optimization of the performance might be possible through adjustment of the parameters. A comparison of “standard” and optimized circuits, in addition, could allow us to estimate the relative quality of the automatic design algorithm. To assess these aspects, we focus on the least complex circuit scheme for test case A (solution 1). For instance, to obtain more reliable experimental measurements, we can aim at improving the circuit performance by enhancing the signal separation up to about 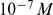. This corresponds to a difference between low and high output signals of approximately 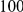 proteins in a typical bacterial cell, which is faithfully detectable by fluorescence microscopy techniques [47].

Using sensitivity analysis, we discovered that only 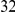 out of the 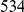 parameters pertaining to the entire network model highly influence the circuit output signal. Since we want to obtain an amplification of the signal separation, we can try to modify only some of the eight sensitive parameters that belong to the final NOR gate. Remarkably, a 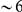-fold increase of the promoter strength produced a clear amplification of the high-level output, while the low-level output was maintained below 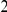 protein copies. Moreover, the new promoter transcription rate is comparable with published data [48] (see Text S1 for details). Hence, while the automatically designed circuits may not be optimally functional, only simple changes may be needed to further improve their performance.

### Circuit robustness

The previous simulations confirmed the validity of our design method because all the alternative designs faithfully reproduced the truth table. In evaluating the performance of automatically designed circuits so far, however, we employed default parameter settings for all parts of the models. The choice of these parameter values has been made starting from published data and, where necessary, by tuning them in order to mimic Boolean gates properly. For instance, data available for riboswitches [49], [50] did not always fit into our model based on full mass-action kinetics. We assumed an effector-aptamer affinity close to the one of the sRNA with its mRNA target site [51] or, without values for these interactions, we referred to the transcription factor-promoter system (for details, see Text S1). Thus, the models' parameter values are associated with uncertainty, and we therefore analyzed the impact of uncertainty on circuit performance.

First, we assessed the network robustness against overall parameter perturbations of the eight optimized solutions of test case A. Specifically, we evaluated if the signal separation remains above a (practically measurable) threshold of 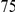 protein copies after randomly modifying all 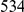 network parameters simultaneously in a range of 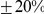 of their reference values. Most of the circuits (six) turned out to be rather robust since more than 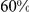 of the corresponding simulations returned a signal separation above this threshold. Hence, uncertainties of parameter values—at least for unstructured parametric perturbations—do not seem to have a great impact on the digital behavior of our networks.

**Figure 7:**
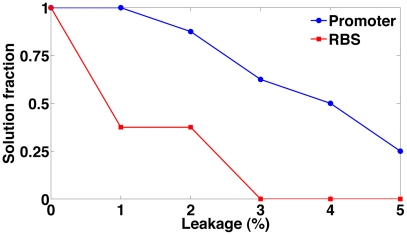
Circuit robustness. Fraction of valid test case A solutions (out of 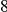) for different promoter and RBS leakage rates. Leakage rate is expressed as a percentage of the fixed transcription/translation rate of the gates that belongs to the circuit internal gates.

From experimental implementations of logical circuits, we know that transcriptional (and translational) leakage can substantially affect circuit performance [27]. As the assumed promoter and RBS basal production rates in our previous analysis were low enough to be negligible, we next simulated test case A solutions with increasing values for the basal transcription and translation rates. As expected, the number of solutions that properly reproduce the circuit truth table decreases by increasing either basal production (Fig. 7). Promoters with a leakage rate of 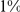 of the internal gate transcription rate–i.e. 10-fold higher than the initial leakage rate–did not affect the qualitative, but the quantitative performance of all 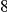 solutions. With further increase of the leakage rate up to the 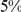 of the reference value, 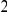 solutions still survive. This might represent a hurdle to the *in vivo* implementation since promoters often have a basal rate of this order of magnitude. Hence, promoters might require several modifications before being used for digital circuit implementation. Analogous conclusions hold also for the RBS. In fact, with translation leakage at 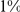 of the internal gate translation rate, the number of valid solutions drops immediately to 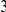 and none of them shows digital behavior with higher basal protein production. Hence, also efficient RBSs are crucial to build reliable digital circuits. Overall, the differences in sensitivity between design alternatives can be employed for prioritizing practical circuit implementations, effectively using robustness as a screening metric.

Besides leakage, another source of signal disturbance is given by the intrinsic random fluctuations present in every biological system [52]. The robustness of a circuit to intrinsic noise can be evaluated via stochastic simulations. Since such simulations are rather time consuming, we examined only the optimized version of test case A, solution 1. Remarkably, this circuit shows a high robustness to intrinsic noise; the signal separation of about 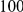 proteins achieved in the deterministic case decreases–within a stochastic framework–only to about 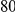 proteins at steady state (see Text S1). This underlines the importance of optimizing the circuit performance by an amplification of the circuit output that makes the 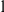 signals insensitive to fluctuations of the 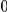 signals and therefore preserves a reasonable separation between both. Overall, thus, the automatically designed circuits are remarkably resilient to model uncertainties and stochastic noise, except for factors such as transcriptional and translational leakage.

## Discussion

We presented a procedure for the automatic design of digital synthetic gene circuits based on standard biological parts. It borrows the Karnaugh map method from electrical engineering to translate a given truth table into a circuit scheme. Fundamentally, the design method employs a set of (models of) biological parts and Boolean gates that allow circuit composition. In our *in silico* implementation, circuits can have up to four different inputs (chemicals) and a single output such as a reporter protein. For each scheme, several circuit designs of different structural complexity and response quality are generated. We characterized circuit performance through output signal separation at steady state and via initial transients and found good solutions for all our simulations.

The main distinction of our method from other currently available computational tools for automatic circuit design is that the circuit structure is computed without the need for any optimization procedure. Optimization as employed in [7], [8] generally is highly compute intensive, especially because it requires simulation of the circuit behavior for all input combinations at each evaluation of the optimization function. In addition, we developed more detailed models for translation—in contrast to the one-step representation in [7], [8]. This implies that translational controls at the mRNA level can be exploited (alone or together with promoter regulation) to assemble Boolean gates. Both aspects lead to a high diversity of possible circuit designs for each truth table generated by our tool within computational times of only few seconds.

In terms of computational complexity, note that deriving a minimal Boolean formula from a truth table is an NP-complete problem. The traditional Karnaugh map algorithm is known to be efficient only up to 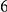 inputs. For more complex systems with a higher number of inputs or outputs, however, logic synthesis in electronic circuit design provides by far more efficient algorithms than the Karnaugh map method [53]. Here, we refrained from corresponding implementations because it was not clear *a priori* if the electronics approach could be transferred to biological systems, and because implementation is the main bottleneck for synthetic gene circuits; already the complexity of some of our example circuits is beyond the limits of the biological digital circuits so far implemented *in vivo*.

As another limitation, our model requires more, potentially ill-characterized or not measurable parameters to specify all the reactions taking place inside every part. However, different gates may share identical parts and inside a part, some parameters share the same value (like the five different mRNA decay rates in a composite RBS). As a consequence, the *net* number of parameters necessary to fully specify a circuit may drop considerably. In test case A solution 1, for instance, only 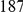 out of 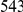 parameters need specific assignments. Among them, interactions between transcription factors and promoters are already well-described and quantitative data are available in the literature. Bigger uncertainties characterize the translational controls we adopted in our framework; we tuned several associated parameter values to assure reasonable parts and gate performance. Hence, improvements to our riboswitch and antisense RNA descriptions are desirable, but to a certain extent the resulting digital circuits are robust to parameter perturbations and intrinsic noise. Moreover, the circuit performance can be drastically improved by acting only on parameters belonging to the final gate.

We want to stress that the importance of our tool lies in the rational, automatic design of complex digital circuits with three or four inputs. As previously explained, basic (two-input) gates have been found in several biological systems. We do not aim to reproduce these rather simple gates but we use them as standard bricks to construct more complex circuits whose structure cannot be designed in an ad-hoc fashion like other small synthetic gene circuits. So far, we considered only a limited number of transcription and translation controls to mimic Boolean behavior. We plan to extend our tool by other regulatory mechanisms already used for *in vivo* implementations of synthetic Boolean gates such as RNA interference [23], tRNA-mRNA base-pairing [21], ribozymes [33], and antiswitches [43].

In terms of practical implementations, even though many of the circuits designed by our tool seem still too large to be implemented *in vivo*, recent progress in combinatorial generation of standard components and their model-based assembly into synthetic circuits [54] suggests that increasingly complex networks will be feasible–and realized–in the near future. In particular, exponentially growing capacities for DNA synthesis [55] and innovative protocols for the simultaneous cloning of multiple parts into a single vector [56], [57] substantially increase our experimental capabilities. Also original, powerful designs for RNA-based Boolean gates [33], and new computational tools for the automated design of each category of Standard Biological Parts such as the RBS calculator [58] will be critical factors in these developments. This will enable novel synthetic signal processing capabilities for applications as biosensors [15], [16] and as molecular diagnostic computers [17].

## Materials and Methods

### Logical gates

Promoter-based configurations [24] are available for every biological gate controlled by one or two inputs: NOT and NOR gates require simple repressions; YES and AND gates simple activation (although, in our implementation, activators act always cooperatively on AND-like promoters); OR gates demand synergistic activation of transcription (by two activators), and, finally, NAND gates need cooperativity between two repressors. Simple activation and repression can be achieved also on the RBS either via sRNA base-pairing with specific target sequences or by chemical binding to a riboswitch. Hence, AND, YES, NOR, and NOT gates can entirely lie on mRNA. On the contrary, since ribosomes are not recruited by sRNAs, we cannot model OR gates on the RBS. Furthermore, NAND gates can be realized only by tandem riboswitches inhibited by the cooperative binding of two effector molecules but never via sRNA regulation that does not provide any kind of cooperativity. For details on the implementation of all gate variants in the three circuit layers we refer to the Text S1.

### Circuit design

We implemented the algorithm for automatic circuit design in Perl. It reads the number of input signals and the truth table of the circuit from a text file. Then, it computes and ranks all the possible circuit solutions, which are logged in a text file. To implement a specific solution, the program asks for the one to be designed. Then, all the necessary biological parts are generated and joined into gate-devices. Devices and pools are subsequently connected to each other to finalize the circuit design. We assign default values (see Text S1) to the kinetic parameters in order to reproduce correct Boolean behaviors. Our tool creates MDL (Model Definition Language [35]) files that fully specify single parts, devices and the whole circuit. They serve as inputs for ProMoT, where the circuit can be visualized and, if needed, parameter values can be changed. Furthermore, ProMoT allows to export the circuit code into formats suitable for simulations such as Matlab (MathWorks, Nantucket/MA) and SBML [59].

### Circuit analysis

Since the model of standard biological parts and pools is fully based on mass-action kinetics (see [4] and Text S1), circuit time-dependent behavior can be simulated both through deterministic and stochastic solvers. For our deterministic simulations, we used the (slightly modified) Matlab codes generated by ProMoT: they are called by another Matlab script that permits to obtain all the entries of a truth table within a single run. The Matlab files for test case A (solution 1) are provided as Protocols S1, S2. We performed our stochastic simulations with COPASI [60] that contains an implementation of the Gibson-Bruck algorithm [61]. The SBML file (level 2, version 1) for test case A (solution 1) is provided as Protocol S3.

Sensitivity analysis and kinetic parameter value optimization were performed in Matlab. In particular, we calculated the normalized sensitivity values of the circuit output (fluorescent protein concentration) with respect to all the circuit kinetic parameters at steady state conditions.

As for the parameter optimization, we used the “gaSB” function (standard genetic algorithm with elitism [62]) and the “Manual Tuning” option of the SBPD extension package for the SBtoolbox2 [63] for Matlab (version 7.8).

## Supporting Information

Figure S1Different three-layer implementations of an XOR gate in electronics. (A) SOP representation: AND-OR and NAND-NAND logic. (B) POS representation: OR-AND and NOR-NOR logic. In both cases, one logic can be derived from the other one through De Morgan's laws: 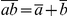 and 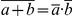.(0.21 MB TIF)

Figure S2 Schemes of YES (A) and NOT (B,C) input gates both in SOP and POS representation. Notice that the standard biological part (promoter or RBS) where the gate output acts is shown at the bottom-right corner of each gate.
(0.05 MB PDF)

Protocol S1 Matlab file - test case A, solution 1.
(0.18 MB TXT)

Protocol S2 Matlab file - test case A, solution 1 simulation.
(0.00 MB TXT)

Protocol S3 SBML file - test case A, solution 1.
(0.45 MB XML)

Text S1 Automatic design of digital synthetic gene circuits. Additional background, methods, and results.
(2.77 MB PDF)
