# Supplementary figures and images for: Automatic Design of Digital Synthetic Gene Circuits
Source: PLoS Comput Biol. 2011 Feb 17;7(2):e1001083. doi: 10.1371/journal.pcbi.1001083 (PMC3048778; doi:10.1371/journal.pcbi.1001083)

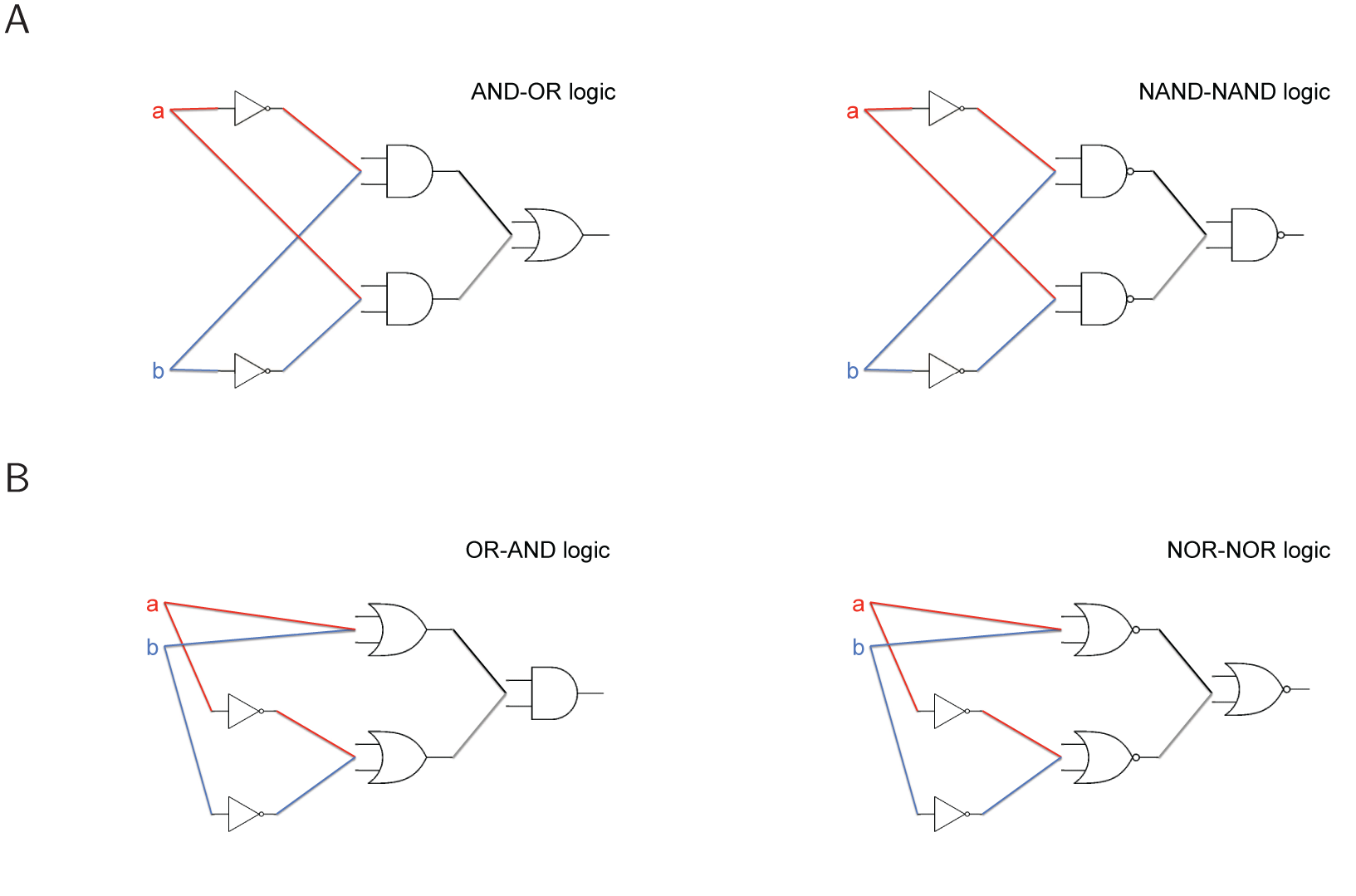

Supplement: Figure S1 — Different three-layer implementations of an XOR gate in electronics. (A) SOP representation: AND-OR and NAND-NAND logic. (B) POS representation: OR-AND and NOR-NOR logic. In both cases, one logic can be derived from the other one through De Morgan's laws: and . (0.21 MB TIF) [file pcbi.1001083.s001.tif]

A

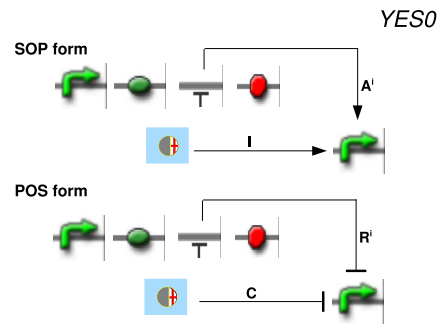

B

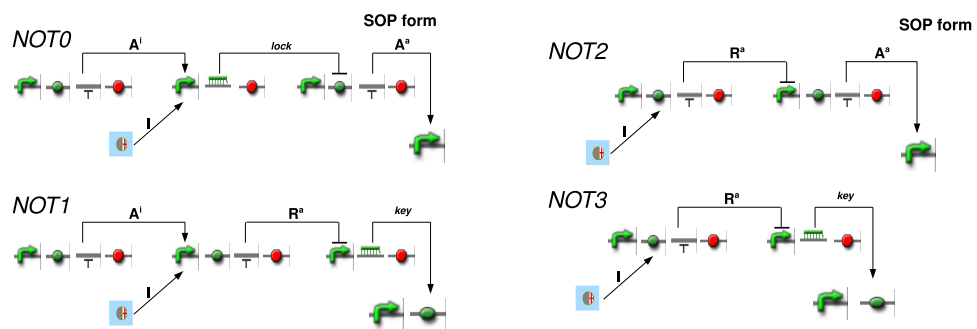

C

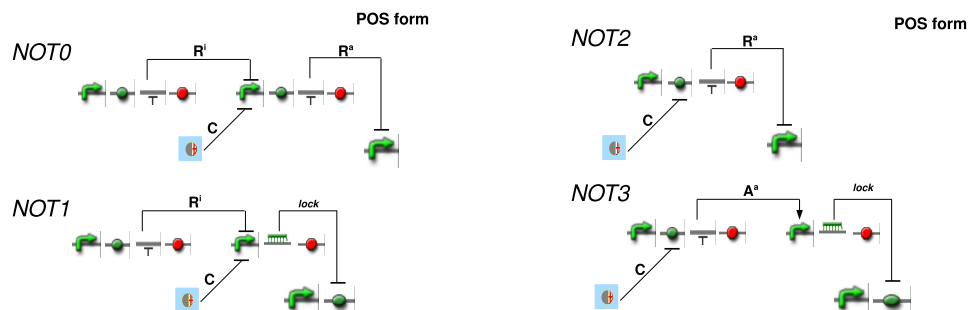

Supplement: Figure S2 — Schemes of YES (A) and NOT (B,C) input gates both in SOP and POS representation. Notice that the standard biological part (promoter or RBS) where the gate output acts is shown at the bottom-right corner of each gate. (0.05 MB PDF) [file pcbi.1001083.s002.pdf]
