# Supplementary material for: Automatic Design of Digital Synthetic Gene Circuits
Source: PLoS Comput Biol. 2011 Feb 17;7(2):e1001083. doi: 10.1371/journal.pcbi.1001083 (PMC3048778; doi:10.1371/journal.pcbi.1001083)
Supplement: Text S1 — Automatic design of digital synthetic gene circuits. Additional background, methods, and results. (2.77 MB PDF) [file pcbi.1001083.s006.pdf]

Automatic design of digital synthetic gene circuits.
Supporting Information.

M.A. Marchisio and J. Stelling

Contents

1 Notions of electronics **2**
1.1 Boolean gates: the truth tables 2
1.2 The Karnaugh map method 3

2 Parts, pools, and gene circuits. **7**

3 RBS model **8**
3.1 Symbols 8
3.2 Inducible/repressible RBSs 8
3.3 Inducible/repressible RBS examples 11
3.3.1 Constitutive RBS 11
3.3.2 One b-site RBS activated by an inducer 11
3.3.3 Two b-site RBS repressed by a corepressor and a lock 12

4 Alternative solutions **15**
4.0.4 Disjoint solutions 15
4.0.5 Mixed and compact solutions 16

5 Implementation of logical gates **17**
5.1 Gate analysis 18
5.1.1 Input gates 18
5.1.2 Internal and final gates 18

6 Circuit simulations **20**
6.1 Test case A 20
6.2 Test case B 22
6.3 Test case C 24
6.4 Test case D 26

7 Sensitivity analysis and circuit optimization **28**

8 Parameter values **31**
8.1 Common values 31
8.1.1 Promoters 31
8.1.2 RBSs 31
8.2 Input gates 32
8.2.1 Promoters 32
8.2.2 RBS 33
8.3 Internal and final gates 33
8.3.1 Promoters 33
8.3.2 RBS 34

9 Additional tables **35**

Chapter 1

Notions of electronics

1.1 Boolean gates: the truth tables

A	NOT
0	1
1	0

A	B	XOR
0	0	0
0	1	1
1	0	1
1	1	0

A	B	AND
0	0	0
0	1	0
1	0	0
1	1	1

A	B	NAND
0	0	1
0	1	1
1	0	1
1	1	0

A	B	OR
0	0	0
0	1	1
1	0	1
1	1	1

A	B	NOR
0	0	1
0	1	0
1	0	0
1	1	0

Figure 1.1: Truth tables of some basic Boolean gates.

1.2 The Karnaugh map method

The Karnaugh map method is an efficient algorithm to determine the *minimal* Boolean formula that corresponds to a given truth table. It requires four steps:

1. converting the truth table into a Karnaugh map;
2. grouping the ones or the zeros inside the Karnaugh map;
3. deriving the logical clause corresponding to each group of ones or zeros;
4. writing the complete Boolean formula.

A Karnaugh map is nothing else than a particular rearrangement of the truth table. The logical variables are displayed both on the rows and on the columns of a Karnaugh map and their values are written in a *Gray code* sequence i.e. two successive values differ only for one bit (see Figure 1.2.

Truth table

a	b	c	d	O
0	0	0	0	0
0	0	0	1	0
0	0	1	0	0
0	1	0	0	1
1	0	0	0	0
0	0	1	1	0
0	1	0	1	1
1	0	0	1	1
0	1	1	0	1
1	0	1	0	1
1	1	0	0	1
0	1	1	1	0
1	0	1	1	0
1	1	0	1	1
1	1	1	0	0
1	1	1	1	0

→

Karnaugh map

cd / ab	00	01	11	10
00	0	0	0	0
01	1	1	0	1
11	1	1	0	0
10	0	1	0	1

Figure 1.2: Rearranging a truth table into a Karnaugh map.

With the Karnaugh map method, it is possible to calculate two Boolean formulas for any given truth table: one in Conjunctive Normal Form (CNF or POS, Product of Sum) and the other in Disjunctive Normal Form (DNF or SOP, Sum of Products). The former requires to group the zeros inside the Karnaugh map, the latter the ones.
Let us consider the truth table and its Karnaugh map in Figure 1.2 and derive the two corresponding Boolean formulas. As stated previously, the DNF requires to group all the ones inside the Karnaugh map. Each group must contain a power of two of 1s. Hence, looking at our Karnaugh map we see that the possible size of the groups will be: 1, 2, and 4. At the beginning, we should gather as many 1s as possible into group of four elements. Afterwards, the remaining 1s should be put into group of size 2 (overlaps between groups are permitted). Eventually, if some 1s remain isolated from all the groups, they will give rise to separate groups of just one element (see Figure

1.3). Each group corresponds to a clause whose length (the number of logical variables inside the clause) is inversely proportional to the size of the group. Therefore, the described "grouping" procedure minimizes both the number and the length of the clauses.

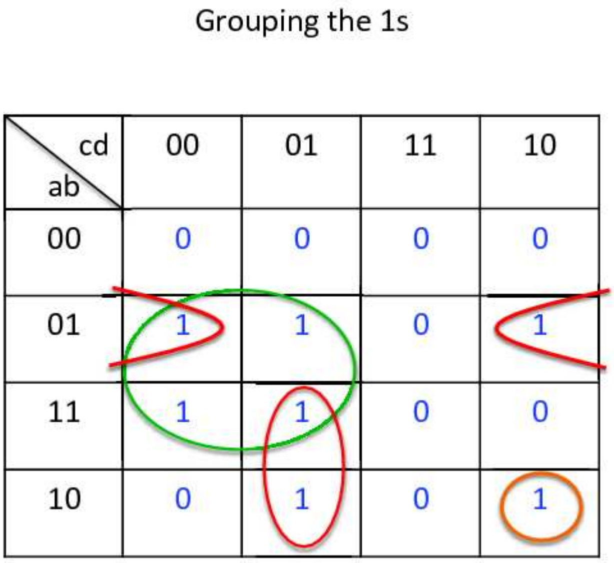

Figure 1.3: Groups of 1s inside the Karnaugh map.

Overall, we have got four different groups: one of 4 elements, two of 2 elements, and one of just one element. Let us consider the only group of size 4 in our Karnaugh map. In order to determine the Boolean clause associated with it, we have, first, to compare the corresponding entries in the truth table:

a	b	c	d
0	1	0	0
0	1	0	1
1	1	0	0
1	1	0	1
X	1	0	X

Table 1.1: Clause corresponding to the group of four ones.

From the last line in the Table 1.1, we see that only b and c conserve their bit. Hence, the clause arising from this group will be given by the product of b (whose value is 1) times $\overline{c}$ (since the value of c is 0).
After obtaining the first clause $(b \cdot \overline{c})$, we can compute the remaining three in a similar way. The group of size 2 on the second line of the Karnaugh map corresponds to $(\overline{a} \cdot b \cdot \overline{d})$ since:

a	b	c	d
0	1	0	0
0	1	1	0
0	1	X	0

The other group of size 2 (on the second column of the Karnaugh map) generates the clause $(a \cdot \overline{c} \cdot d)$, which follows from:

a	b	c	d
1	1	0	1
1	0	0	1
1	X	0	1

The last group, which contains only one element, corresponds to a unique truth table entry (1010) that is translated directly into the clause $(a \cdot \overline{b} \cdot c \cdot \overline{d})$.
Finally, the DNF Boolean formula is given by the sum of these four clauses:

$$(b \cdot \overline{c}) + (\overline{a} \cdot b \cdot \overline{d}) + (a \cdot \overline{c} \cdot d) + (a \cdot \overline{b} \cdot c \cdot \overline{d})\,. \tag{1.1}$$

Let us derive the second Boolean formula (in CNF) that describes our starting truth table. The procedure is similar to the one followed above but, this time, we have to group the zeros inside the Karnaugh map as shown in Figure 1.4

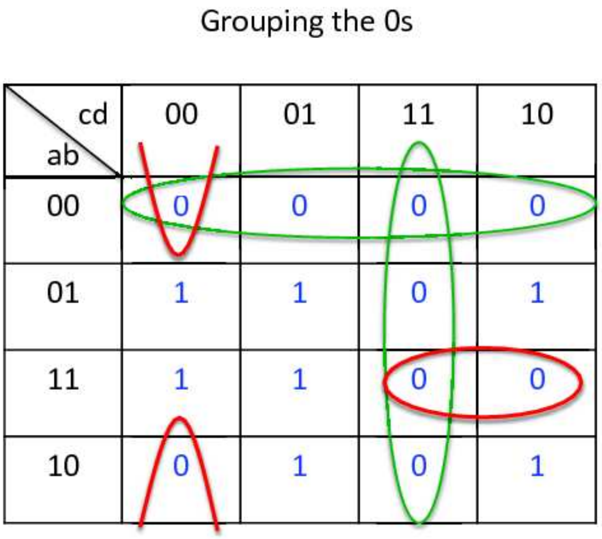

Figure 1.4: Groups of 0s inside the Karnaugh map.

We have got four groups again: two of size 4 and the other two of size 2.
In the group given by the first row of our Karnaugh map, the values of a and b are conserved:

a	b	c	d
0	0	0	0
0	0	0	1
0	0	1	1
0	0	1	0
0	0	X	X
1	1	X	X

Table 1.2: Clause corresponding to the row of four zeros.

Since–differently from DNF–in CNF we have to consider the complement of the conserved bits to write a clause properly (see Table 1.2), the group we are examining generates the clause $(a + b)$. Now, it is straightforward to compute the clause of the other group of size 4: $(\overline{c} + \overline{d})$, as well as of the group of size 2 in the first column of the Karnaugh map: $(b + c + d)$ and of the group of the same size in the third row: $(\overline{a} + \overline{b} + \overline{c})$.

Overall, the CNF Boolean formula of our truth table is the product of the clauses we have just computed:

$$(a + b) \cdot (\overline{c} + \overline{d}) \cdot (b + c + d) \cdot (\overline{a} + \overline{b} + \overline{c})\,. \tag{1.2}$$

From both formulas, a scheme of an electronic circuit organized into three layers of gates follows straightforwardly. Each variable that is negated demands a NOT gate in the first layer; every clause corresponds either to an AND (DNF) or an OR (CNF) gate in the second layer. The third layer is, finally, given either by an OR (DNF) or an AND (CNF) gate that gathers the binary outputs coming from the gates placed in the second layer.
Both formulas, in the above example example, require 9 gates for a circuit implementation: 4 NOTs, 4 AND/OR (second layer), and one OR/AND (third layer). Therefore, both are minimal. Notice, however, that as explained in the main text the concept of minimal formula is in biology different from the one in electronics. As a consequence, the way the groups are defined into a Karnaugh map–irrelevant in electronics–may determine circuit schemes of different complexity in biology. To illustrate this differences consider for instance the SOP formulas: $(\overline{c} \cdot \overline{d}) + (c \cdot d) + (a \cdot b \cdot \overline{c})$ and $(\overline{c} \cdot \overline{d}) + (c \cdot d) + (a \cdot b \cdot d)$. They represent the same truth table and, in electronics, they give rise to the same circuit consisting of 6 gates. In biology, on the contrary, the former expression–as a best solution–furnishes a circuit made of 8 genes with a complexity score $S = 5$, whereas the latter requires 9 genes and a more complex structure ($S = 6$).

Chapter 2

Parts, pools, and gene circuits.

Standard biological parts are DNA traits associated with well-defined functions. To design Boolean gates we need only a subset of these parts: promoters, where RNA polymerase binds and transcription starts; ribosome binding sites (RBS), the place where translation begins; coding regions, which contain the sequence for proteins (transcription factors or reporters) and for small antisense RNAs; terminators.
Pools are an abstraction and represent the place where free molecules of *common signal carriers* are stored. In our model, we take into account five kinds of signal carriers: RNA polymerases, ribosomes, small RNAs, transcription factors, and chemicals (environmental signals). In circuit design, pools are the interface among devices (transcription units) or between the whole circuit and the cellular environment. For more information, see [16].

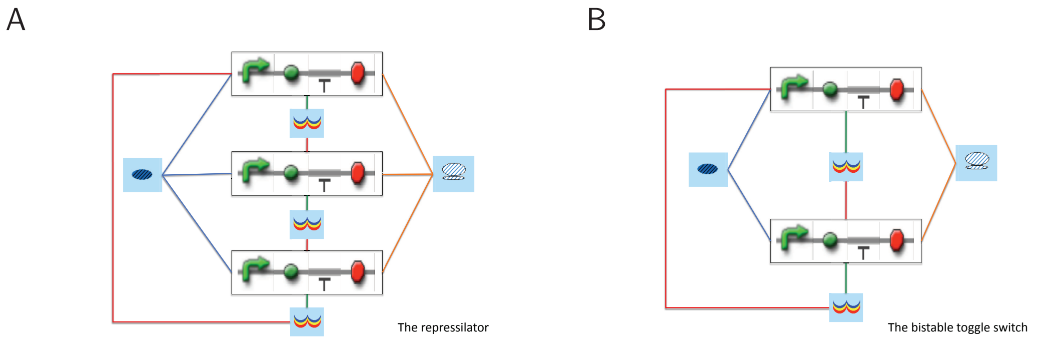

Figure 2.1: The repressilator [5] (A) and the bistable toggle switch [8] (B) by means of standard biological parts and pools. Line meaning: blue, flux of RNA polymerases; orange, flux of ribosomes; green, flux of transcription factors from transcription units to pools; red, flux of transcription factor from pools to transcription units.

Chapter 3

RBS model

The RBS is a complex part that can be controlled by up to two regulatory factors. They bind to specific mRNA sequences–here indicated as *b sites*–which are the analogous of the promoter operators. We focused our attention on the RBSs that can behave–either alone or together with the promoter–as logic gates and we developed a library of 17 possible combinations.

3.1 Symbols

B	free DNA RBS
$[PolB]$	RNA polymerase-RBS complex
Pol^{el}	RNA polymerases in the elongation phase
$b_{1,2}^{n,f}$	b sites
$[rb_{1,2}^{n}]$	ribosome-RBS complex
r^{free}	free ribosomes
r^{cl}	ribosomes in the clearing phase
$PoPS^{out}$	Polymerase Per Second - output flux to a protein coding
$PoPS^{lk}$	Polymerase Per Second due to leakage - input flux from a leaky promoter
$PoPS^{rt}$	Polymerase Per Second due to readthrough - input flux from a terminator
$RiPS^{out}$	Ribosomes Per Second - output flux to a protein coding
$RiPS^{lk}$	Ribosomes Per Second due to RBS leakage - output flux to a protein coding
$SiPS^{b}$	Signals Per Second - flux exchanged with a signal pool
$RNAPS^{b}$	RNA Per Second - flux exchanged with a small RNA pool
k_{el}	RNA polymerase elongation rate constant
v_{el}	RNA polymerase elongation velocity
l_{RBS}	RBS lenght
t_{cl}^{r}	ribosomal clearence time

3.2 Inducible/repressible RBSs

An RBS can be regulated by effector molecules (like thiamine and tetracycline) that act on riboswitches or by sRNAs that bind to their complementary RNA sequences. We refer to the effectors as *inducers* (I) and *corepressors* (C) in analogy to the promoter; sRNAs are denoted as *keys* (k, activator-like) or *locks* (l, repressor-like). Every b-site can lie in two states: *on* and *off*, allowing and preventing ribosome binding, respectively.
RBSs can be divided into three classes:

1. constitutive RBS: it does not contain any b-site for regulatory factors so that ribosomes can bind without any constraint;

2. one b-site RBSs: they exist in four configurations, two repressible (bound by a lock or by a corepressor) and two inducible (bound by a key or by an inducer);

3. two b-site RBSs: the most complex case. It has been modeled according to the following rules:

   - cooperativity behavior (homo and hetero) is possible only between effectors (see for instance [15]);
   - two b-sites hosting the same effector molecules are allowed (mimicking a class of tandem riboswitches); b-sites for sRNAs are on the contrary supposed to have different sequences;
   - when an effector and an sRNA are hosted inside an RBS, by convention the effector binds to the b_1 site and the sRNA to the b_2 one;
   - a configuration where a b-site is activated and the other repressed by the corresponding regulatory factors is not allowed (it is not relevant for logic behavior).

Basal production

If at least one b-site is present inside the RBS, the protein synthesis is increased by a factor due to the ribosome leakage. Every RBS state containing at least one *off* b-site contributes to the basal protein production with a term proportional to its own concentration times the leakage rate constant. The generated $RiPS^{lk}$ flux is sent to the next protein coding where it increases the total protein concentration.

Incoming $PoPS^{rt}$ and $PoPS^{lk}$

When present, the $PoPS^{rt}$ coming from the next terminator and the $PoPS^{lk}$ arriving from the previous promoter increase either an *off* or an *on* state depending of the kind of regulatory factors acting on the RBSs. Inducers and keys bind to b-sites in the *off* states, whereas corepressors and locks to the ones that are *on* by default.

Translation: basic ideas

Let us consider the simplest RBS configuration, the constitutive one. RNA polymerase incoming from the promoter (as $PoPS^{in}$) binds to a site B, which belongs to this part, giving rise to the complex $[PolB]$. Then, it starts $mRNA$ transcription, moving along at the elongation velocity (v_{el}), mantained also inside the next protein coding part (although this model does not force polymerase to have the same velocity inside these two parts).
As long as the leader is transcribed, ribosomes can bind to the mRNA at the site b of the RBS (in the proximity of the Shine-Dalgarno sequence) forming the $[rb]$ complex. After clearing this region, ribosomes are imagined to join the AUG codon (which belongs to the protein coding part) where they form another complex ($[ra]$) that coincides with the translation starting point.
As the promoter is the generator of the $PoPS$ signal, the RBS can be seen as the generator of the $RiPS$ signal.
The RBS can be, furthermore, connected to the terminator placed at the end of the transcription unit. From here, a $PoPS^{rt}$ signal is received and it is used to estimate the expression of the adjacent coding region inside the next transcription unit due the readthrough phenomenon. Although this is a $PoPS$ signal, it modifies the amount of mRNA here transcribed.
A similar contribution is given by another polymerase flux, $PoPS^{lk}$, which comes from the adjacent promoter.
It is assumed that readthrough effects involve only two successive transcription units. This follows from consideration about the average ribosome elongation velocity, the gene length and the mRNA decay rate constant.

**Site number**	**Regulatory Factors**	**Interactions**
none	none	(consitutive RBS)
1	I_1, C_1, l_1, K_1	
2	$l_1 l_2$, $C_1 l_2$, $C_1 C_2$, $C_1 C_1$	none
2	$C_1 C_2$, $C_1 C_1$	cooperativity
2	$k_1 k_2$, $I_1 k_2$, $I_1 I_2$, $I_1 I_1$	none
2	$I_1 I_2$, $I_1 I_1$	cooperativity

Table 3.1: Possible RBS configurations. They depend on the regulatory factors and on their interactions.

**RBS states**	
b_1^n	state 1 (one b-site RBS, n stands for *on*)
b_1^f	state 2 (one b-site RBS, f stands for off)
$b_1^n b_2^n$	state 1 (two b-site RBS)
$b_1^n b_2^f$	state 2 (two b-site RBS)
$b_1^f b_2^n$	state 3 (two b-site RBS)
$b_1^f b_2^f$	state 4 (two b-site RBS)
**k_d decay rate constants**	
k_{di}	i refers to the RBS state
k_{dr1}	degradation of the ribosome-RBS (state 1) complex
k_{db}	degradation of free mRNA in a constitutive RBS
k_{drb}	degradation of the ribosome-RBS complex in a constitutive RBS
**θ and ξ ribosomal rate constants**	
θ_{is}	i refers to the b-site where a regulatory factor binds to; s to the state (on or off) of the other b-site (if present)
ξ_{is}	i refers to the b-site which a regulatory factor is leaving; s to the state (on or off) of the other b-site (if present)
**k_r ribosomal Michaelis-Menten kinetic constants**	
k_{1r} and k_{-1r}	1 and -1 indicate interaction between ribosomes and RBS
k_{2r}	2 indicates "translation";
**k_r^{lk} leakage rate constants**	
k_{rs}^{lk}	s indicates the RBS state

Table 3.2: General notation adopted in this chapter.

3.3 Inducible/repressible RBS examples

3.3.1 Constitutive RBS

Reactions

$$PoPS^{in} \Longrightarrow [PolB] \xrightarrow{k_{el}} Pol^{el} + B \quad (3.1)$$

$$PoPS^{rt} \Longrightarrow b \quad (3.2)$$

$$PoPS^{lk} \Longrightarrow b \quad (3.3)$$

$$r^{free} + b \overset{(k_{1r},k_{-1r})}{\rightleftharpoons} [rb] \xrightarrow{k_{2r}} r^{cl} + b \quad (3.4)$$

$$b \xrightarrow{k_{db}} \quad (3.5)$$

$$[rb] \xrightarrow{k_{drb}} r^{free} \quad (3.6)$$

Equations

$$k_{el} = \frac{v_{el}}{l_{RBS}} \quad (3.7)$$

$$\frac{d[PolB]}{dt} = PoPS^{in} - k_{el}[PolB] \quad (3.8)$$

$$PoPS^{out} = k_{el}[PolB] \quad (3.9)$$

$$k_{2r} = (t^r_{cl})^{-1} \quad (3.10)$$

$$\frac{db}{dt} = k_{el}[PolB] - k_{1r}r^{free}b + k_{-1r}[rb] + k_{2r}[rb] - k_{db}b + PoPS^{rt} + PoPS^{lk} \quad (3.11)$$

$$\frac{d[rb]}{dt} = k_{1r}r^{free}b - k_{-1r}[rb] - k_{2r}[rb] - k_{drb}[rb] \quad (3.12)$$

$$RiPS^{b} = -k_{1r}r^{free}b + k_{-1r}[rb] + k_{drb}[rb] \quad (3.13)$$

$$RiPS^{out} = k_{2r}[rb] \quad (3.14)$$

$$RiPS^{lk} = 0 \quad (3.15)$$

Comments

It holds that

$$k_{drb} = k_{db}$$

3.3.2 One b-site RBS activated by an inducer

This RBS is *off* by default. I_1 turns b_1^f into b_1^n, allowing ribosome binding.

Reactions

$$PoPS^{in} \Longrightarrow [PolB] \xrightarrow{k_{el}} Pol^{el} + B \tag{3.16}$$

$$PoPS^{rt} \Longrightarrow b_1^f \tag{3.17}$$

$$PoPS^{lk} \Longrightarrow b_1^f \tag{3.18}$$

$$I_1 + b_1^f \overset{(\theta_1,\xi_1)}{\rightleftharpoons} b_1^n \tag{3.19}$$

$$r^{free} + b_1^n \overset{(k_{1r},k_{-1r})}{\rightleftharpoons} [rb_1^n] \overset{k_{2r}}{\rightarrow} r^{cl} + b_1^n \tag{3.20}$$

$$b_1^n \xrightarrow{k_{d1}} I_1 \tag{3.21}$$

$$b_1^f \xrightarrow{k_{d2}} \tag{3.22}$$

$$[rb_1^n] \xrightarrow{k_{dr1}} r^{free} + I_1 \tag{3.23}$$

Equations

$$k_{el} = \frac{v_{el}}{l_{RBS}} \tag{3.24}$$

$$\frac{d[PolB]}{dt} = PoPS^{in} - k_{el}[PolB] \tag{3.25}$$

$$PoPS^{out} = k_{el}[PolB] \tag{3.26}$$

$$k_{2r} = (t_{cl}^r)^{-1} \tag{3.27}$$

$$\frac{db_1^n}{dt} = \theta_1 I_1 b_1^f - \xi_1 b_1^n - k_{1r} r^{free} b_1^n + k_{-1r}[rb_1^n] + k_{2r}[rb_1^n] - k_{d1} b_1^n \tag{3.28}$$

$$\frac{db_1^f}{dt} = k_{el}[PolB] - \theta_1 I_1 b_1^f + \xi_1 b_1^n - k_{d2} b_1^f + PoPS^{rt} + PoPS^{lk} \tag{3.29}$$

$$\frac{d[rb_1^n]}{dt} = k_{1r} r^{free} b_1^n - k_{-1r}[rb_1^n] - k_{2r}[rb_1^n] - k_{dr1}[rb_1^n] \tag{3.30}$$

$$RiPS^b = -k_{1r} r^{free} b_1^n + k_{-1r}[rb_1^n] + k_{dr1}[rb_1^n] \tag{3.31}$$

$$RiPS^{out} = k_{2r}[rb_1^n] \tag{3.32}$$

$$RiPS^{lk} = k_{r2}^{lk} b_1^f \tag{3.33}$$

$$SiPS^b = -\theta_1 I_1 b_1^f + \xi_1 b_1^n + k_{d1} b_1^n + k_{dr1}[rb_1^n] \tag{3.34}$$

3.3.3 Two b-site RBS repressed by a corepressor and a lock

This RBS is *on* by default. C_1 turns b_1^n into b_1^f and l_2 turns b_2^n into b_2^f.

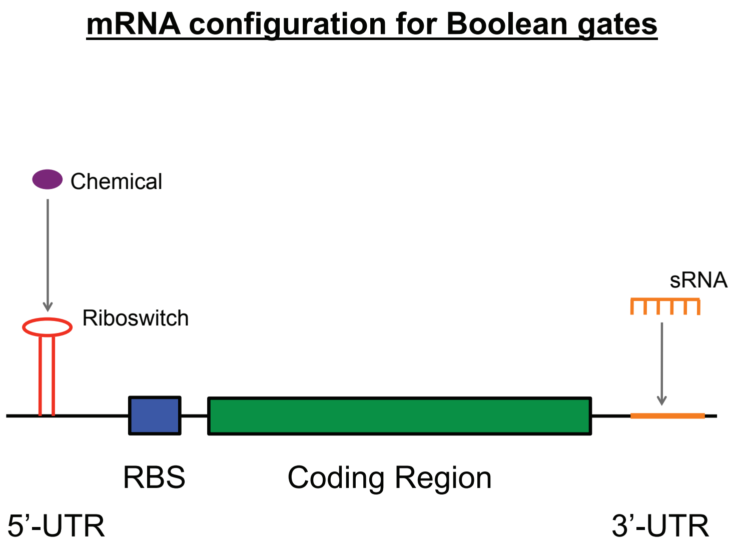

Figure 3.1: Possible configuration of an mRNA-based Boolean gate controlled via both riboswitch/chemical interaction and sRNA/mRNA base-pairing. Riboswitches are commonly placed along the 5' UTR (un-translated region) whereas the sRNA binding site lie on the 3'-UTR. In our model, both controls have been inserted into the RBS part with no loss of generality, however.

Reactions

$$PoPS^{in} \Longrightarrow [PolB] \xrightarrow{k_{el}} Pol^{el} + B \tag{3.35}$$

$$PoPS^{rt} \Longrightarrow b_1^n b_2^n \tag{3.36}$$

$$PoPS^{lk} \Longrightarrow b_1^n b_2^n \tag{3.37}$$

$$C_1 + b_1^n b_2^n \overset{(\theta_1^n, \xi_1^n)}{\rightleftharpoons} b_1^f b_2^n \tag{3.38}$$

$$C_1 + b_1^n b_2^f \overset{(\theta_1^f, \xi_1^f)}{\rightleftharpoons} b_1^f b_2^f \tag{3.39}$$

$$l_2 + b_1^n b_2^n \overset{(\theta_2^n, \xi_2^n)}{\rightleftharpoons} b_1^n b_2^f \tag{3.40}$$

$$l_2 + b_1^f b_2^n \overset{(\theta_2^f, \xi_2^f)}{\rightleftharpoons} b_1^f b_2^f \tag{3.41}$$

$$r^{free} + b_1^n b_2^n \overset{(k_{1r}, k_{-1r})}{\rightleftharpoons} [r b_1^n b_2^n] \overset{k_{2r}}{\to} r^{cl} + b_1^n b_2^n \tag{3.42}$$

$$b_1^n b_2^n \xrightarrow{k_{d1}} \tag{3.43}$$

$$b_1^n b_2^f \xrightarrow{k_{d2}} \tag{3.44}$$

$$b_1^f b_2^n \xrightarrow{k_{d3}} C_1 \tag{3.45}$$

$$b_1^f b_2^f \xrightarrow{k_{d4}} C_1 \tag{3.46}$$

$$[rb_1^n b_2^n] \xrightarrow{k_{dr1}} r^{free} \tag{3.47}$$

Equations

$$k_{el} = \frac{v_{el}}{l_{RBS}} \tag{3.48}$$

$$\frac{d[PolB]}{dt} = PoPS^{in} - k_{el}[PolB] \tag{3.49}$$

$$PoPS^{out} = k_{el}[PolB] \tag{3.50}$$

$$k_{2r} = (t_{cl}^r)^{-1} \tag{3.51}$$

$$\frac{db_1^n b_1^n}{dt} = k_{el}[PolB] - \theta_1^n C_1 b_1^n b_2^n + \xi_1^n b_1^f b_2^n - \theta_2^n l_2 b_1^n b_2^n + \xi_2^n b_1^n b_2^f + \tag{3.52}$$

$$- \quad k_{d1} b_1^n b_2^n - k_{1r} r^{free} b_1^n b_2^n + k_{1r}[rb_1^n b_2^n] + k_{2r}[rb_1^n b_2^n] + PoPS^{rt} + PoPS^{lk} \tag{3.53}$$

$$\frac{db_1^n b_1^f}{dt} = \xi_1^f b_1^f b_2^f - \theta_1^f C_1 b_1^n b_2^f + \theta_2^n l_2 b_1^n b_2^n - \xi_2^n b_1^n b_2^f - k_{d2} b_1^n b_2^f \tag{3.54}$$

$$\frac{db_1^f b_1^n}{dt} = \theta_1^n C_1 b_1^n b_2^n - \xi_1^n b_1^f b_2^n \xi_2^f b_1^f b_2^f - \theta_2^f l_2 b_1^f b_2^n - k_{d3} b_1^f b_2^n \tag{3.55}$$

$$\frac{db_1^f b_1^f}{dt} = \theta_1^f C_1 b_1^n b_2^f - \xi_1^f b_1^f b_2^f + \theta_2^f l_2 b_1^f b_2^n - \xi_2^f b_1^f b_2^f - k_{d4} b_1^f b_2^f \tag{3.56}$$

$$\frac{d[rb_1^n b_2^n]}{dt} = k_{1r} r^{free} b_1^n b_2^n - k_{-1r}[rb_1^n b_2^n] - k_{2r}[rb_1^n b_2^n] - k_{dr1}[rb_1^n b_2^n] \tag{3.57}$$

$$RiPS^b = -k_{1r} r^{free} b_1^n b_2^n + k_{-1r}[rb_1^n b_2^n] + k_{dr1}[rb_1^n b_2^n] \tag{3.58}$$

$$RiPS^{out} = k_{2r}[rb_1^n b_2^n] \tag{3.59}$$

$$RiPS^{lk} = k_{r2}^{lk} b_1^n b_1^f + k_{r3}^{lk} b_1^f b_1^n + k_{r4}^{lk} b_1^f b_1^f \tag{3.60}$$

$$RNAPS^b = \xi_2^n b_1^n b_2^f - \theta_2^f l_2 b_1^f b_2^n + \xi_2^f b_1^f b_2^f - \theta_2^n l_2 b_1^n b_2^n \tag{3.61}$$

$$SiPS^b = \xi_1^n b_1^f b_2^n + \xi_1^f b_1^f b_2^f - \theta_1^n C_1 b_1^n b_2^n - \theta_1^f C_1 b_1^n b_2^f + k_{d3} b_1^f b_2^n + k_{d4} b_1^f b_2^f \tag{3.62}$$

Chapter 4

Alternative solutions

4.0.4 Disjoint solutions

Circuit schemes that are a direct representation of a Boolean formula show a unique final gate that collects the signals coming from the gates in the internal layer (single class solutions). Beside this kind of circuits, we constructed also other more complex schemes with a different final layer design. As explained in the main text, these alternative solutions have been used to determine if a better performance can be achieved by increasing the structural complexity of our "basic" schemes.
First, we took into account other single gates solutions that differ from each other by the number of regulatory factors acting on the final layer gate (an example is shown in Figure 4.1B). Then, we required that each internal gate produces a different regulatory factor (*disjoint* solutions), which implies that the final layer needs to be split into two sublayers, since a single gate cannot take more than four different inputs (two proteins and two sRNAs). For POS, the first sublayer is made of OR gates and the second one consists of a NOR gate (*OR-NOR disjoint*–see Figure 4.1C). Alternatively, the first sublayer can be made of NOR gates whose inputs are gathered by a final AND gate (*NOR-AND disjoint*) like in Figure 4.1D. SOP final schemes for disjoint solutions are built on the POS ones with the inclusion of a NOT gate after the final NOR/AND. When the final AND gets only two inputs, it is directly converted into a NAND.

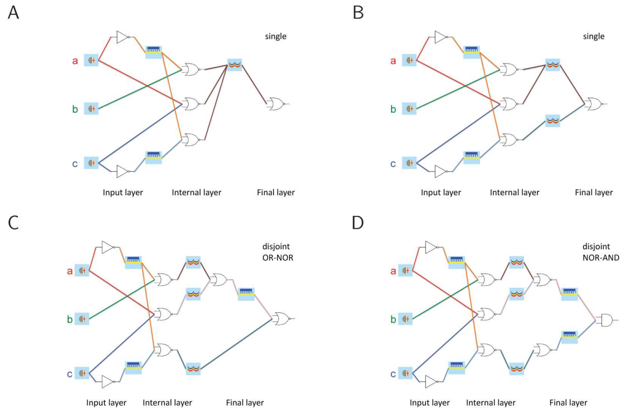

Figure 4.1: Layered circuit structure. Gene digital circuits exhibit a layered structure. Chemicals act on the first two layers whereas the final layers can have several configurations. Here, the same circuit is built with four different final layers designs: (A) and (B) *single* gate fed with one and two transcription factors, respectively; (C) an OR gate converts two internal-gate outputs into an input (sRNA) for the final NOR gate; (D) two NOR gates are placed between the internal layer and the final AND gate.

4.0.5 Mixed and compact solutions

Particular Boolean formulas give rise, on the contrary, to solutions that are less complex than the *single* class ones. In fact, when a formula in POS has at least one clause that contains a unique, negated literal, it is convenient to send the corresponding input signal directly to the RBS of the final gate in order to save two gates in the first two layers. In SOP, on the contrary, the input signal is sent to a NOT2 input gate whose product (an activator) binds the final gate promoter without the need for a YES, redundant gate in the internal layers. These kind of solutions have been named *compact*.
Another possibility is given by the conversion of some rather simple formulas into circuits that employ inducers and co-repressors as inputs in a *mixed* solution. For example, the expression $(a+b)\cdot\overline{c}\cdot\overline{d}$ could use an OR promoter controlled by two activators joint to a NOR RBS made of a tandem riboswitch repressed by two chemicals. This circuit requires only 3 genes (two YES gates plus the "mixed" gate), instead of the 6 genes for a pure POS solution. The formulas that show a mixed solution are listen in Table 4.1.

Formula	Promoter logic	RBS logic
$(a+b)\cdot c$	OR	YES
$(a+b)\cdot\overline{c}$	OR	NOT
$(\overline{a}+\overline{b})\cdot c$	NAND	YES
$(\overline{a}+\overline{b})\cdot\overline{c}$	NAND	NOT
$(a+b)\cdot c\cdot d$	OR	AND
$(a+b)\cdot\overline{c}\cdot\overline{d}$	OR	NOR
$(\overline{a}+\overline{b})\cdot c\cdot d$	NAND	AND
$(\overline{a}+\overline{b})\cdot\overline{c}\cdot\overline{d}$	NAND	NOR

Table 4.1: Formulas suitable for a mixed solution.

Chapter 5

Implementation of logical gates

We can realize a digital circuit by plugging together biological Boolean gates defined in our library. If they faithfully reproduce their own truth tables, the overall circuit will work properly. Notably, only few parameters determine the digital behavior of a biological device. The most significant parameters are: the affinities of the promoter/RBS for their regulatory factors in comparison with the RNA polymerase/ribosome binding and dissociation rate, the promoter/RBS strength, and the regulatory factors' (including small-molecule inputs') half lives [12, 26, 2, 21].

Promoter and RBS leakage play important roles in determining the overall circuit performance as well, and we studied their effects separately (see section "Circuit robustness" in the main text). We defined a parametric reference point by keeping the RBS strength, the regulatory factor decay rates and all the parameters that describe the RNA polymerase and ribosome kinetics constant. Subsequently, we tuned the regulatory factor dynamics. Here, we qualitatively depict the rules for finalizing a digital gene circuit design; numerical values are provided in the next section.

NOR and NOT gates require a full, rapid repression of the transcription and translation activity: this can be obtained by setting the promoter-repressor/RBS-lock affinity much higher than the promoter-RNA polymerase/RBS-ribosome affinity. In AND gates, on the contrary, there is no competition between regulatory factors and RNA polymerases or ribosomes. Activator and key affinity for their binding sites just need to be sufficiently strong (roughly equal to RNA polymerase and ribosome affinities) to trigger transcription and translation, respectively. AND promoters, furthermore, require cooperativity of activators. Internal YES gates are designed in the same way but they contain only a single binding site either for an activator or for a key.

OR gates, controlled by activators as well, can be realized with the same affinity values for the AND promoters. Furthermore, synergistic activation of transcription has to be turned on. In order to determine the promoter strengths, we have to assure that the first two layers produce regulatory factors in a quantity either sufficient to repress/activate the gates in the next layer (1 output) or very close to zero (0 output). Furthermore, when the gate output is a protein (transcription factor or reporter), the internal and final gate promoters should be weak. In fact, on one hand, AND and OR gates must not produce a significant amount of molecules when the input signals are very low (0 input). On the other hand, when a circuit is switched on–chemicals are sent to the first two layers–the NOR (and the AND) gates that should be inactive can produce a transient output before the circuit reaches the steady state. To reduce the intensity and the duration of the transient output, NOR promoters have to be weak as well. For uniform output production of the internal and final layer gates, we chose a unique value for the promoter strength. In contrast, if small RNAs have to be transcribed, promoters have to be rather strong because sRNAs decay faster than proteins and their production is not amplified by translation.

In particular, we set the maximal transcription rate for proteins to $0.5s^{-1}$ [5]. Then, we fixed the promoter strength to one tenth ($0.05s^{-1}$) and to the double ($1.0s^{-1}$) of this value for all the gates that release proteins and sRNAs, respectively. With these parameter values, every fully induced internal/final gate–present in a single plasmid–produces, when inserted into a circuit, ≈ 4 mRNAs for roughly 27 repressors or $10 \div 27$ activators (reporter proteins can be more numerous

because they are not supposed to dimerize). Otherwise, about 300 locks or more than 200 keys are transcribed at the steady state. Furthermore, the 0 output corresponds to less than one transcription factor (about 0.1 repressors and 0.05 activators) and two sRNAs (≈ 0.5 locks and ≈ 1.3 keys). Therefore, at steady state, the regulatory factor quantity is much bigger or clearly lower than the amount of DNA and mRNA binding sites and, with an opportune choice of affinities values (see above), every internal and final gate can be activated and repressed properly. As for the input layer, NOT gates are generally made of two or three genes in a cascade. Therefore, to get a fast conversion of a chemical into a protein, we placed strong promoters (transcription rate: $0.5s^{-1}$) in their first transcription units. Moreover, the produced proteins/sRNAs are the inputs for up to five different promoters/RBSs (four in the internal layer and one belonging to the same NOT gate). Then, as 1 output, more than 5 proteins and more than 20 sRNAs have to be synthesized by a NOT gate. The above considerations imply that this can be achieved by using the same parameter values as in the internal/final gates.
Overall, we achieved functional designs of synthetic digital gene circuits by using simple considerations about mRNA, DNA and regulatory factor concentrations, and about the mechanisms of repression and activation of standard biological parts. For sake of simplicity, we modeled the RBS regulation mechanisms and promoter control identically. In terms of parameter values, we did not distinguish between the interactions of sRNAs and chemicals with mRNA. However, better knowledge of mRNA kinetics and dynamics may be necessary to improve the reliability of our tool as a guide for wet lab experiments. A detailed analysis of the gates' performance (inside a circuit) is given in the next section.

5.1 Gate analysis

5.1.1 Input gates

The structures of all the input gates are shown in Figure S3. In the tables below, the name of each gate is accompanied by the input signal (i, inducer; c, corepressor) and by the output (R, repressor; A, activator; l, lock; k, key). Signal separation is calculated–for every gate–at steady state condition ($t = 20000\,s$).

Name	σ (M)	ρ (%)	Transient
NOT0c_R	$2.66E-08$	0.01	815.49
NOT1c_l	$3.29E-07$	0.00	430.64
NOT2c_R	$2.66E-08$	0.00	776.04
NOT3c_l	$3.22E-07$	0.09	3975.39

Table 5.1: Gate performance - POS

Name	σ (M)	ρ (%)	Transient
NOT0i_A	$2.73E-08$	0.43	1239.08
NOT1i_k	$3.32E-07$	0.20	1057.73
NOT2i_A	$2.72E-08$	0.35	1363.11
NOT3i_k	$3.35E-07$	0.13	778.01

Table 5.2: Gate performance - SOP

5.1.2 Internal and final gates

The name of every internal and final gate is followed by the number of inputs ($1 \div 4$), their kind (R,l,c for POS; A,k,i for SOP), and the output type (where f represents fluorescent proteins).

Name	σ (M)	ρ (%)	Transient
NOT1R_R	$2.62E-08$	0.01	0.00
NOT1R_l	$2.91E-07$	0.02	0.00
NOT1l_R	$2.72E-08$	0.07	0.00
NOT1c_R	$2.75E-08$	0.00	832.19
OR2AA_R	$2.41E-08$	0.00	817.20
OR2AA_l	$1.25E-07$	0.03	1767.76
NOR2RR_R	$2.73E-08$	0.37	118.80
NOR2RR_l	$3.46E-07$	0.15	106.73
NOR2ll_R	$2.68E-07$	0.02	982.05
NOR2Rl_R	$2.72E-08$	0.38	27.90
NOR2Rc_R	$2.73E-08$	0.39	0.00
NOR2lc_R	$2.73E-08$	0.18	0.00
NOR2cc_R	$2.75E-08$	0.00	819.04
NOR3RRl_R	$2.71E-08$	0.37	18.32
NOR3Rcc_R	$2.73E-08$	0.39	18.87
NOR3Rll_R	$2.71E-08$	0.38	15.06
NOR3RRc_R	$2.73E-08$	0.37	2.58
NOR3Rlc_R	$2.72E-08$	0.38	12.15
NOR4RRlc_R	$2.71E-08$	0.37	8.63
NOR4RRll_R	$2.70E-08$	0.36	5.27
NOR4RRcc_R	$2.73E-08$	0.37	1.23

Table 5.3: Gate performance - POS

Name	σ (M)	ρ (%)	Transient
YES1A_A	$2.57E-08$	0.21	3044.41
YES1A_k	$2.98E-07$	0.43	2118.90
YES1k_A	$1.80E-08$	0.00	1503.85
YES1i_A	$2.63E-08$	0.00	0.00
OR2AA_R	$1.65E-08$	0.00	0.00
OR2AA_l	$1.31E-07$	0.02	0.00
NAND2RR_f	$6.24E-08$	0.89	0.00
AND2AA_A	$2.16E-08$	0.09	2429.70
AND2AA_k	$2.06E-07$	0.22	1782.75
AND2Ak_A	$1.67E-08$	0.17	2008.16
AND2Ai_A	$2.45E-08$	0.20	390.81
AND2kk_A	$1.34E-08$	0.00	1435.93
AND2ik_A	$1.78E-08$	0.00	63.12
AND2ii_A	$2.67E-08$	0.00	0.00
AND3AAk_A	$1.41E-08$	0.07	1873.49
AND3AAi_A	$2.06E-08$	0.09	308.84
AND3Akk_A	$1.25E-08$	0.15	1654.74
AND3Aik_A	$1.66E-08$	0.17	234.26
AND3Aii_A	$2.50E-08$	0.21	188.78
AND4AAkk_A	$1.04E-08$	0.06	1601.49
AND4AAki_A	$1.39E-08$	0.07	200.45
AND4AAii_A	$2.10E-08$	0.09	165.28

Table 5.4: Gate performance - SOP

Chapter 6

Circuit simulations

6.1 Test case A

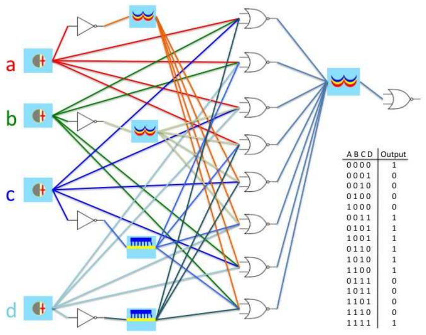

Figure 6.1: Solution1 - POS, single

$$
\begin{aligned}
\text{POS} &= (a+b+c+\overline{d})\cdot(a+b+\overline{c}+d)\cdot(a+\overline{b}+c+d)\cdot(a+\overline{b}+\overline{c}+\overline{d}) \\
&\quad \cdot(\overline{a}+\overline{b}+c+\overline{d})\cdot(\overline{a}+\overline{b}+\overline{c}+d)\cdot(\overline{a}+b+c+d)\cdot(\overline{a}+b+\overline{c}+\overline{d}) \\
\text{SOP} &= (\overline{a}\cdot\overline{b}\cdot\overline{c}\cdot\overline{d})+(\overline{a}\cdot\overline{b}\cdot c\cdot d)+(\overline{a}\cdot b\cdot\overline{c}\cdot d)+(\overline{a}\cdot b\cdot c\cdot\overline{d}) \\
&\quad +(a\cdot b\cdot\overline{c}\cdot\overline{d})+(a\cdot b\cdot c\cdot d)+(a\cdot\overline{b}\cdot\overline{c}\cdot d)+(a\cdot\overline{b}\cdot c\cdot\overline{d})
\end{aligned}
$$

Solution	Score	σ (nM)	ρ (%)	Transient
1 (POS, single)	20	36.0	3.02	3709.27
25 (SOP, single)	22	58.3	7.71	5707.65
2 (POS, single)	36	33.6	3.19	3641.08
26 (SOP, single)	38	31.2	1.37	3912.29
4 (POS, OR-NOR)	548	62.1	2.72	5112.28
27 (SOP, NOR-AND)	1062	44.1	5.46	6272.92
3 (POS, OR-NOR)	2060	27.3	4.69	5545.99
28 (SOP, NOR-AND)	2062	43.9	2.53	6333.21

Table 6.1: Circuit performance

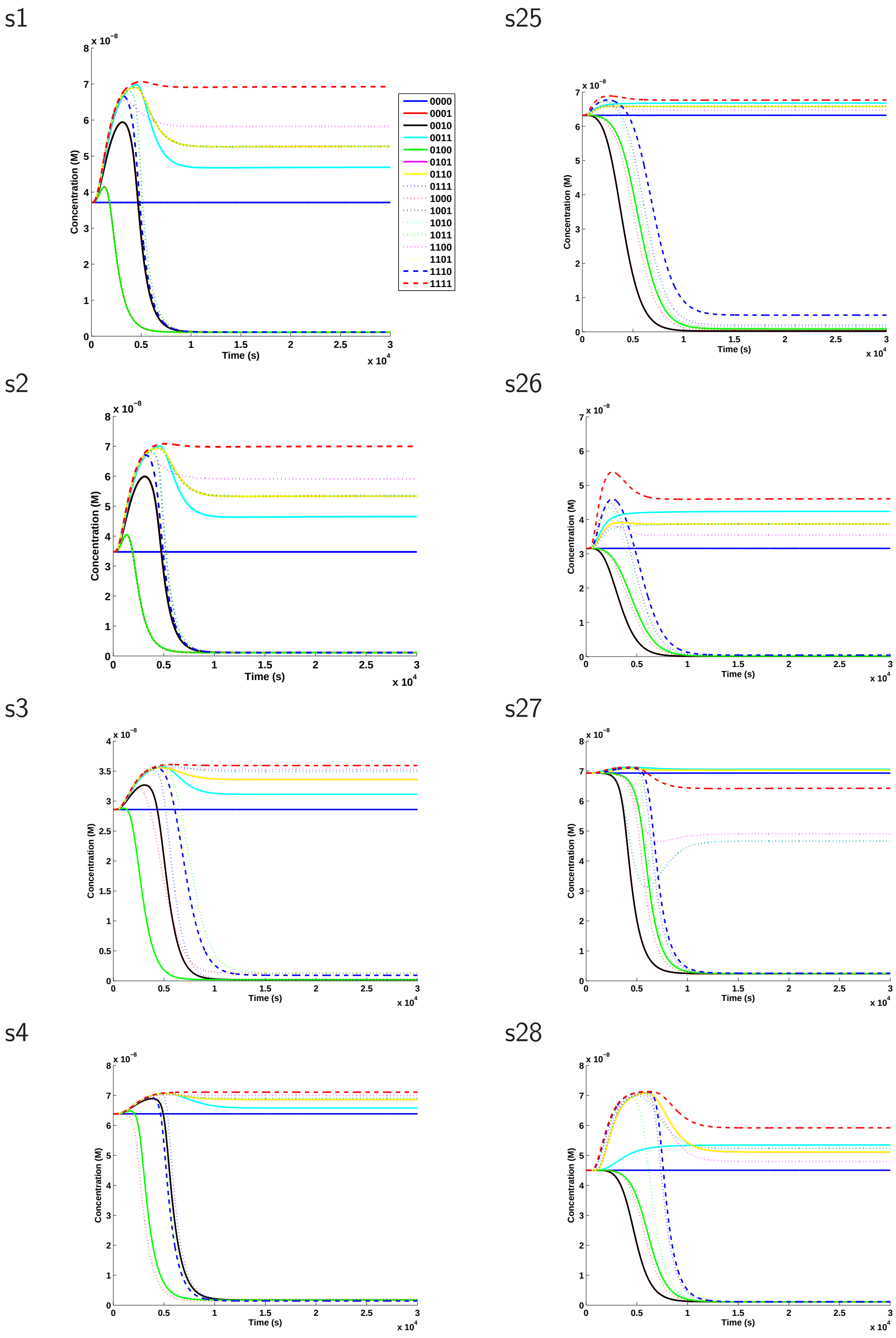

Figure 6.2: Simulations

6.2 Test case B

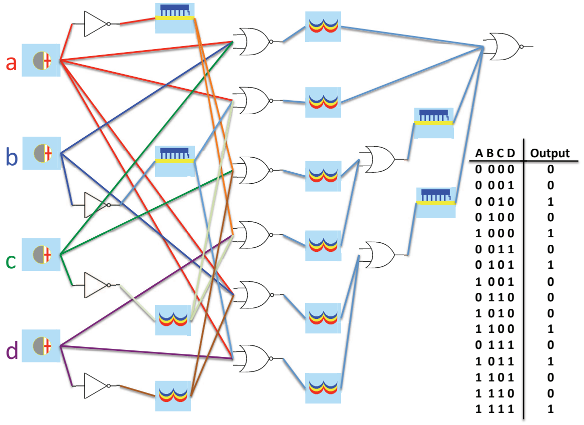

Figure 6.3: Solution166 - POS, OR-NOR disjoint

$$\begin{aligned} \text{POS} &= (a+b+c)\cdot(a+\overline{b}+\overline{c})\cdot(\overline{a}+c+\overline{d})\cdot(\overline{a}+\overline{c}+d)\cdot(a+b+\overline{d})\cdot(a+\overline{b}+d) \\ \text{SOP} &= (a\cdot\overline{c}\cdot\overline{d})+(a\cdot c\cdot d)+(\overline{a}\cdot\overline{b}\cdot c\cdot\overline{d})+(\overline{a}\cdot b\cdot\overline{c}\cdot d) \end{aligned}$$

Solution	Score	σ (M)	ρ (%)	Transient)
97 (POS, single)	11	$4.16E-08$	2.64	542.51
93 (POS, single)	11	$4.09E-08$	2.69	1115.81
276 (SOP,single)	15	$6.17E-08$	4.09	2699.50
100 (POS, OR-NOR)	36	$6.01E-08$	2.32	709.84
280 (SOP, OR-NOR)	36	$4.49E-08$	2.63	1025.77
218 (SOP, NOR-AND)	78	$6.75E-08$	2.96	1173.34
95 (POS, NOR-AND)	141	$3.24E-08$	6.13	1621.79
219 (SOP, NOR-NAND)	268	$3.98E-08$	1.25	501.89

Table 6.2: Circuit performance

s97

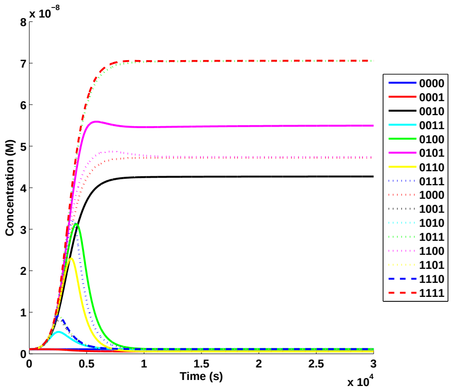

s93

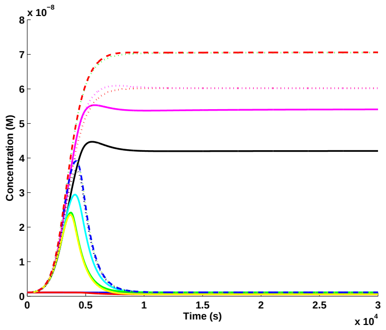

s276

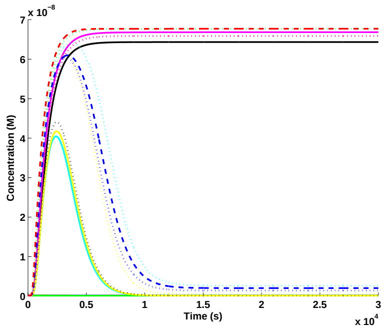

s100

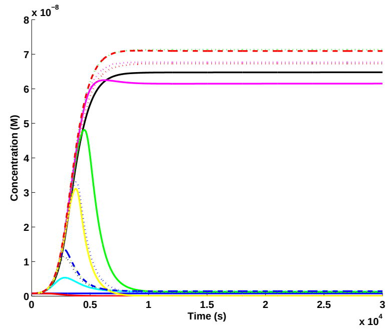

s280

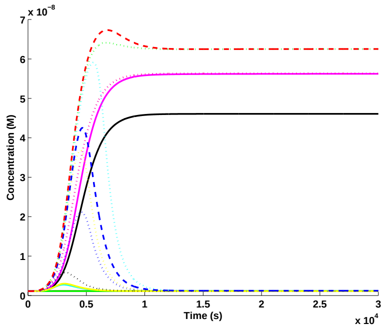

s218

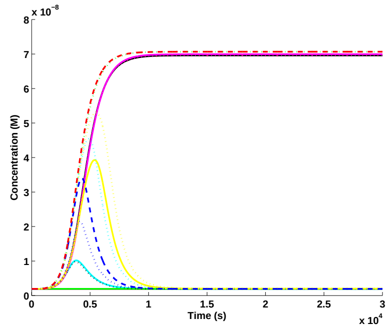

s95

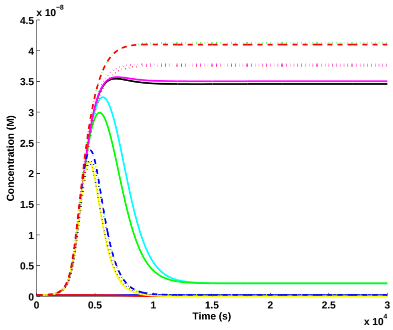

s219

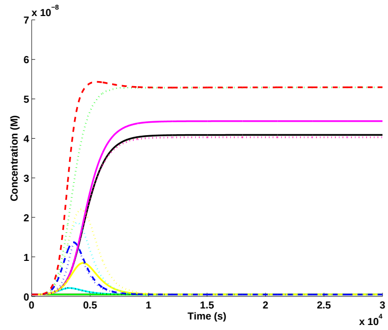

Figure 6.4: Simulations

6.3 Test case C

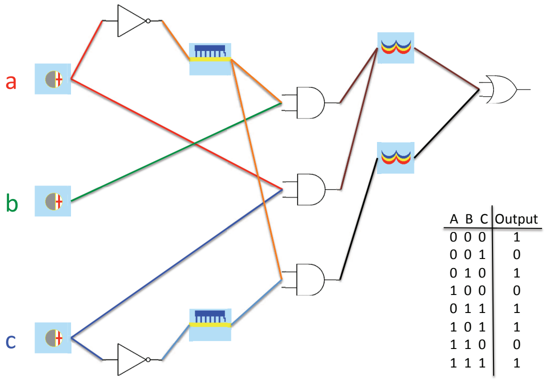

Figure 6.5: Solution166 - SOP, single

$$\text{POS} = (\overline{a} + c) \cdot (a + b + \overline{c})$$
$$\text{SOP} = (\overline{a} \cdot b) + (a \cdot c) + (\overline{a} \cdot \overline{c})$$

Solution	Score	σ (M)	ρ (%)	Transient
51 (POS, single)	4	$4.88E - 08$	2.27	2197.63
159 (SOP, single)	5	$6.52E - 08$	1.07	5852.02
54 (POS, single)	6	$5.58E - 08$	2.00	3803.97
166 (SOP, single)	6	$3.56E - 08$	0.23	2585.08
161 (SOP, OR-NOR)	10	$5.11E - 08$	2.18	4608.97
104 (SOP, single)	14	$6.11E - 08$	2.10	4648.21

Table 6.3: Circuit performance

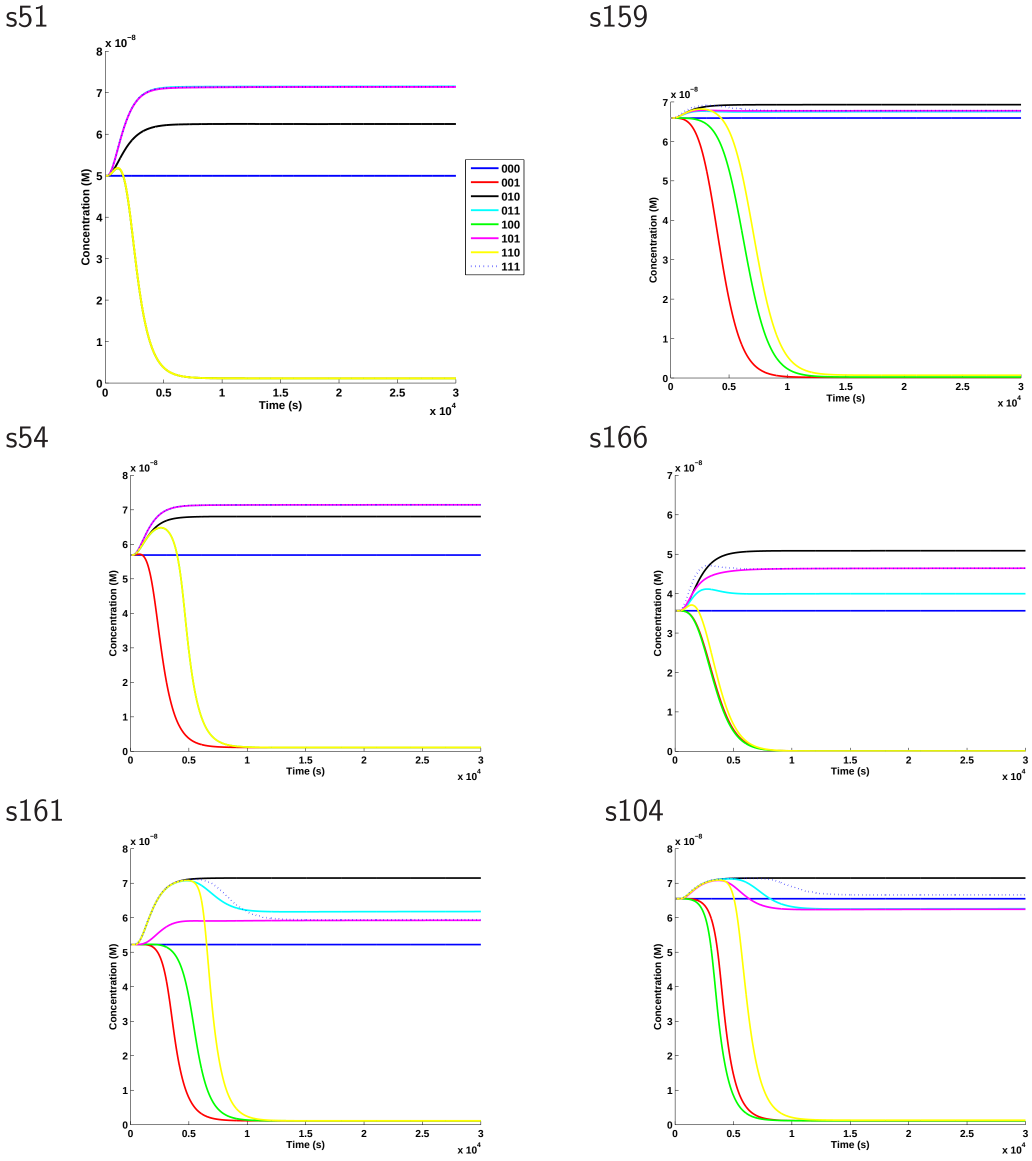

Figure 6.6: Simulations

6.4 Test case D

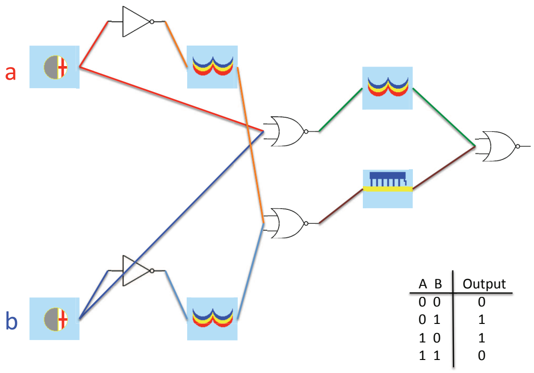

Figure 6.7: Solution 34 - POS, single

$$
\begin{aligned}
\text{POS} &= (a+b)\cdot(\overline{a}+\overline{b}) \\
\text{SOP} &= (\overline{a}\cdot b)+(a\cdot\overline{b})
\end{aligned}
$$

Solution	Score	σ (M)	ρ (%)	Transient
32 (POS, single)	4	$6.09E-08$	1.83	534.93
34 (POS, single)	5	$6.91E-08$	1.60	384.34
75 (SOP, single)	5	$6.58E-08$	0.63	2772.20
80 (SOP, single)	6	$4.02E-08$	0.20	894.73

Table 6.4: Circuit performance

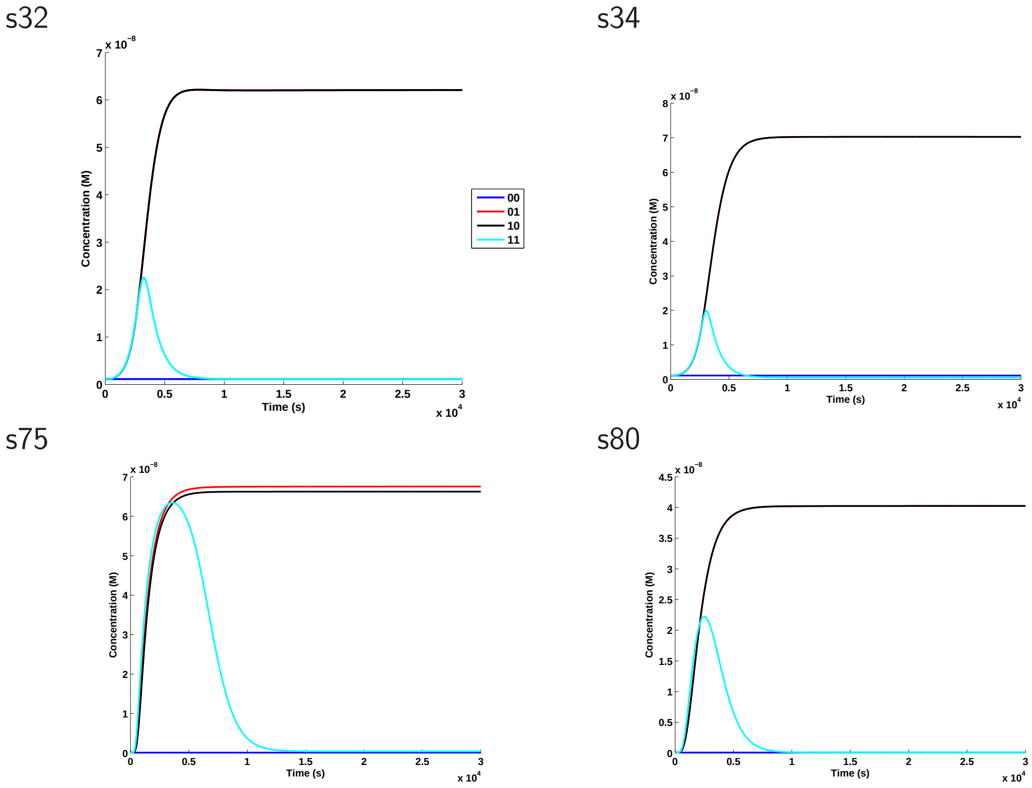

Figure 6.8: Simulations

Chapter 7

Sensitivity analysis and circuit optimization

As reported in the main text, among the 32 parameters that highly influence the circuit output, eight belong to the final NOR gate. The best optimization strategy we figured out required modifying the values of these 8 parameters (see Table 7.1). Furthermore, for simplicity, we kept the default decay rates of the mRNA transcribed from the final NOR gate and of the repressor acting on the final NOR gate. Hence, we were left with 6 parameters to tune in order to achieve the desired absolute signal separation of 100 proteins.
Running an optimization algorithm on the model turned out to be still too time-consuming. This was mainly due to the presence of 16 target functions (the truth table entries). To reduce the computational time, we therefore considered only a subset of the functions, since they are just constant concentrations of fluorescent proteins at steady state (either about $10^{-10}M$ or $10^{-7}M$). Nevertheless, as reported in Table 7.2 and Figure 7.1B, the genetic algorithm we used [22] (on 2, 5 and all the 16 target functions) failed to reproduce these two concentration levels properly. Above all, the 0 output resulted increased up to more than 10 proteins even though the absolute signal separation had doubled with respect to the initial model.
Hence, to our view it was more convenient to ”manually tune” with Matlab (see Circuit Analysis) some of the 6 significant parameters. To this purpose, we used the “Manual Tuning” option of the SBPD extension package for the SBtoolbox2 [20]. This tool provides a graphic user interface where it is possible to select both the model parameters to be optimized and a subset of the truth table entries to which the circuit output should be approximated. After changing the value of any selected parameter, the circuit is newly simulated and its output is plotted against the ”desired” one (represented by constant fluorescent protein concentrations, in our case). Hence, one can see on the computer screen if the previous parameter change improved or not the circuit behavior and, accordingly, perform the next parameter change. This visual optimization procedure is very fast and reliable as long as one wants to modify only a limited number of kinetic parameters, as it was in our case.
As stated in the main text, thanks to this manual tuning operation we realized that we could get to the signal separation of 100 proteins just by increasing the strength of the final gate promoter (k_2).
Furthermore, we performed a longer procedure based on the optimization of each gate’s signal separation (100 proteins, again). The circuit performance reached in this way was very close to the one obtained with our set of default parameter values. This result confirmed the validity of optimizing only parameters belonging to the final gate.
Finally, we run stochastic simulations on both the ”manually” optimized and the non-optimized solution. As shown in Fig. 7.2, circuit optimization assures a signal separation fairly above the minimal detectable threshold of 40 proteins (see the main text).

Parameter	Sensitivity	Part
k_{2r}	1.181	RBS
k_2	1.058	Promoter
k_D	1.051	Coding region
k_d	1.044	RBS
α_1	0.972	Promoter
k_1	0.920	Promoter
β_1	0.824	Promoter
k_D	0.797	Repressor pool

Table 7.1: Sensitivity analysis. Parameters of the final NOR gate that highly influence the concentration of the circuit output (fluorescent protein) at steady state. See [16] for the parameters' symbols.

Name	σ (nM)	max0 (nM)	min1 (nM)	ρ (%)
1	36.0	1.12	37.1	3.02
1_a	40.6	0.07	40.6	0.18
1_m	98.5	1.31	99.9	1.31
1_{2f}	66.1	14.6	80.7	18.09
1_{5f}	69.1	12.6	81.8	15.43
1_{16f}	67.7	14.0	81.7	17.17

Table 7.2: Circuit performance: optimization. Comparison of the output signal separation of test case A solution 1 after different optimization procedures. Names refer to: 1, non-optimized solution; 1_a, optimization on each gate (genetic algorithm and manual tuning); 1_m, manual tuning on the final gate; 1_{2f}, genetic algorithm on the final gate considering 2 target functions; 1_{5f}, genetic algorithm on the final gate considering 5 target functions; 1_{16f} genetic algorithm on the final gate considering all the 16 target functions.

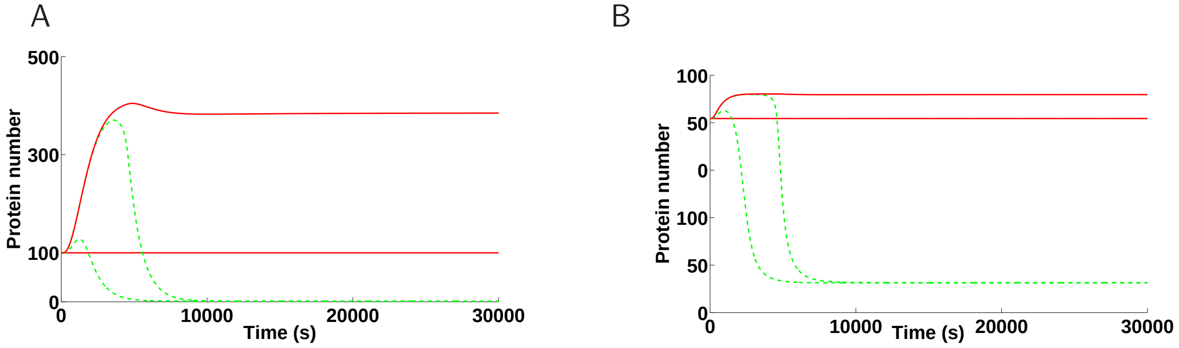

Figure 7.1: Parameter optimization. Simulations of test case A solution 1 after different parameter optimization procedures (1 outputs lie between the red lines, 0 outputs between the green ones). (A) Manual tuning. The circuit output is amplified by increasing only the strength of the promoter belonging to the final NOR gate (k_2). The 0 level is, nevertheless, kept to very low values (see Table 7.2). (B) Genetic algorithm - 5 target functions. Six parameters of the final NOR gate have been optimized through a genetic algorithm [22] to reproduce five entries of the circuit truth table.

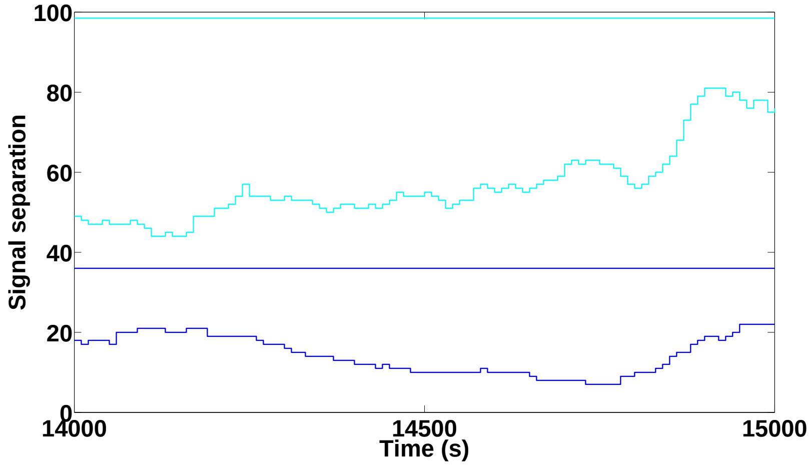

Figure 7.2: Stochastic simulations. Time dependency of the signal separation during stochastic simulation (single run) of both the non-optimized (blue line) and optimized (cyan line) version of test case A solution 1. Continuous lines represent the signal separations obtained from deterministic simulations (36 proteins in the non-optimized case, 98.5 proteins in the optimized one). As apparent from the graphs, circuit optimization is necessary to stay above the detectable threshold of 40 proteins (see main text). All the simulation results were obtained through the COPASI [10] implementation of the Gibson-Bruck algorithm [9].

Chapter 8

Parameter values

As stated in the main text, we chose the same parameter values for the binding of chemicals and sRNAs to the RBS. For more accurate values regarding the riboswitch dynamics see [25] and [18].

8.1 Common values

8.1.1 Promoters

Parameter	Value	Comments
P_T	$1.0 \cdot 10^{-9}\,M$	corresponds to 1 plasmids
k_1	$10^6\,M^{-1}s^{-1}$	[13] and [24] (tuned)
k_{-1}	$0.01\,s^{-1}$	[13] and [24] (tuned)
λ_1	$10^6\,M^{-1}s^{-1}$	[11] (tuned)
μ_1	$0.001\,s^{-1}$	[11] (tuned)
λ_2	$10^6\,M^{-1}s^{-1}$	[11] (tuned)
μ_2	$0.001\,s^{-1}$	[11] (tuned)
k_{D1}	$0.00116\,s^{-1}$	[11]
		(default value for every protein decay rate inside promoter)
k_2^{lk}	$5.0 \cdot 10^{-5}\,s^{-1}$	see main text

8.1.2 RBSs

Parameter	Value	Comments
k_{1r}	$10^6\,M^{-1}s^{-1}$	[4] (tuned)
k_{-1r}	$0.01\,s^{-1}$	[4] (tuned)
k_{2r}	$0.02\,s^{-1}$	[23]
k_{el}	$2\,s^{-1}$	[16]
k_d	$0.0116\,s^{-1}$	[14]
		(default value for every mRNA decay rate inside RBS)
k_{2r}^{lk}	$1.0 \cdot 10^{-5}\,s^{-1}$	see main text

Coding regions

Parameter	Value	Reference
k_D	$0.00116\,s^{-1}$	[11]
k_{el}^{PC}	$0.056\,s^{-1}$	[16]
k_{el}^{r}	$0.049\,s^{-1}$	[16]
ζ_r	$0.5\,s^{-1}$	[7]

sRNAs

Parameter	Value	Reference
k_{el}^{sRNA}	$1.0\,s^{-1}$	corresponds to a length of 40 nt

Terminator

Parameter	Value	Reference
ζ	$31.25\,s^{-1}$	[1]
η	0	

Polymerase pool

Parameter	Value	Reference
$Pol^{free}(t=0)$	$2.1 \cdot 10^{-6}\,M$	[14] (tuned)

Ribosome pool

Parameter	Value	Reference
$r^{free}(t=0)$	$4.2 \cdot 10^{-6}\,M$	[14] and [3] (tuned)

Transcription factor pool

Parameter	Value	Reference
δ	$10^{9}\,M^{-1}s^{-1}$	[24]
ϵ	$10\,s^{-1}$	[24]
k_D	$0.00116\,s^{-1}$	[11]
		(monomers and dimers take the same decay rate value)

sRNA factor pool

Parameter	Value	Reference
k_{dk}	$0.0019\,s^{-1}$	[17]

Signal pool

Parameter	Value	Reference
k	$10^{-9}\,Ms^{-1}$	[16] (tuned)
k_D	$3.21 \cdot 10^{-5}\,s^{-1}$	aTC decay rate

8.2 Input gates

8.2.1 Promoters

Parameter	Value	Comments
k_2	$0.5\,s^{-1}$	strong for protein production [5]
	$0.05\,s^{-1}$	weak for protein production (see Chapter 5)
n_1	0, 1	
α_1	$10^{9}\,M^{-1}s^{-1}$	repressor case [5]
	$10^{6}\,M^{-1}s^{-1}$	activator case - SOP[11] (tuned)
	$10^{5}\,M^{-1}s^{-1}$	activator case - POS [16]
β_1	$0.01\,s^{-1}$	derived from simulations

8.2.2 RBS

Parameter	Value	Comments
θ_1	$10^9\,M^{-1}s^{-1}$	locks and corepressors case
		symmetric to promoter repression
	$10^5\,M^{-1}s^{-1}$	keys and inducers case [6] (tuned)
ξ_1	$0.01\,s^{-1}$	derived from simulations

8.3 Internal and final gates

8.3.1 Promoters

NOT/YES/NOR/AND/OR - one input

Parameter	Value	Comments
k_2	$0.05\,s^{-1}$	weak for protein production (see Chapter 5)
	$1.0\,s^{-1}$	strong for sRNA production (see Chapter 5)
n_1	0, 1	always 0 for final NOR and OR
α_1	$10^9\,M^{-1}s^{-1}$	NOT/NOR gate [5]
	$10^6\,M^{-1}s^{-1}$	YES/OR gate[11] (tuned)
β_1	$0.01\,s^{-1}$	derived from simulations

NOR

Parameter	Value	Comments
k_2	$0.05\,s^{-1}$	weak for protein production (see Chapter 5)
	$1.0\,s^{-1}$	strong for sRNA production (see Chapter 5)
n_1,n_2	0, 1, 2	
α_{1f},α_{1t},α_{2f},α_{2t}	$10^9\,M^{-1}s^{-1}$	[5]
β_{1f},β_{1t},β_{2f},β_{2t}	$0.01\,s^{-1}$	derived from simulations

AND

Parameter	Value	Comments
k_2	$0.05\,s^{-1}$	weak for protein production (see Chapter 5)
	$1.0\,s^{-1}$	strong for sRNA production (see Chapter 5)
n_1,n_2	0, 1	
α_{1f}	$10^3\,M^{-1}s^{-1}$	derived from simulations - cooperativity
α_{1t},α_{2f},α_{2t}	$10^6\,M^{-1}s^{-1}$	[11] (tuned)
β_{1f}	$1\,s^{-1}$	derived from simulations
β_{1t},β_{2f},β_{2t}	$0.01\,s^{-1}$	derived from simulations

OR

Parameter	Value	Comments
k_2	$0.05\,s^{-1}$	weak for protein production (see Chapter 5)
	$1.0\,s^{-1}$	strong for sRNA production (see Chapter 5)
n_1,n_2	0, 1	
α_{1f},α_{1t},α_{2f},α_{2t}	$10^5\,M^{-1}s^{-1}$	[11] (tuned)
β_{1f},β_{1t},β_{2f},β_{2t}	$0.01\,s^{-1}$	derived from simulations
k_{1sa}	$2.1 \cdot 10^6\,M^{-1}s^{-1}$	synergistic activation
k_{-1sa}	$0.0049\,s^{-1}$	synergistic activation
k_{2sa}	$0.11\,s^{-1}$	synergistic activation

NAND

Parameter	Value	Comments
k_2	$0.05\,s^{-1}$	weak for protein production (see Chapter 5)
	$1.0\,s^{-1}$	strong for sRNA production (see Chapter 5)
n_1,n_2	0, 1	
α_{1f},α_{2f}	$10^7\,M^{-1}s^{-1}$	derived from simulations - cooperativity
α_{1t},α_{2t}	$10^9\,M^{-1}s^{-1}$	[11] (tuned)
β_{1f},β_{2f}	$0.1\,s^{-1}$	derived from simulations
β_{1t},β_{2t}	$0.01\,s^{-1}$	derived from simulations

8.3.2 RBS

NOT/YES/NOR/AND - one input

Parameter	Value	Comments
θ_1	$10^9\,M^{-1}s^{-1}$	NOT/NOR case - symmetric to promoter
	$10^5\,M^{-1}s^{-1}$	YES/AND case - [6]
ξ_1	$0.01\,s^{-1}$	derived from simulations

NOR

Parameter	Value	Comments
θ_{1n},θ_{1f},θ_{2n},θ_{2f}	$10^9\,M^{-1}s^{-1}$	symmetric to promoter
ξ_{1n},ξ_{1f},ξ_{2n},ξ_{2f}	$0.01\,s^{-1}$	derived from simulations

AND

Parameter	Value	Comments
θ_{1f},θ_{1t},θ_{2f},θ_{2t}	$10^5\,M^{-1}s^{-1}$	[6] (tuned) - no cooperativity
ξ_{1f},ξ_{1t},ξ_{2f},ξ_{2t}	$0.01\,s^{-1}$	derived from simulations - no cooperativity
θ_{1n},θ_{2n}	$10^6\,M^{-1}s^{-1}$	[6] (tuned) - cooperativity
ξ_{1n},ξ_{2n}	$0.001\,s^{-1}$	derived from simulations - no cooperativity
θ_{1f},θ_{2f}	$10^5\,M^{-1}s^{-1}$	[6] (tuned) - cooperativity
ξ_{1f},ξ_{2f}	$0.01\,s^{-1}$	derived from simulations - no cooperativity

Chapter 9

Additional tables

Circuit	CPU time (s)	Sol. Number	Inputs
NOR	0.039	64	2
XOR	0.040	80	2
NOR	0.147	296	3
XOR	0.138	301	3
NOR	7.173	1616	4
XOR	3.881	1668	4
AND	0.053	101	4
Test Case A	0.054	48	4
Rinaudo	0.078	167	4

Table 9.1: CPU time required to compute all the possible solutions for some circuit examples. Simulations have been run on an Intel CPU Xeon E5440 at 2.83 GHz.

Solution number	Score	A	R	k	l	Gene number	Type
108	2	0	2	0	0	5	POS
57	3	0	2	0	1	6	POS
111	3	1	1	0	1	6	POS
109	4	0	3	0	0	5	POS
54	4	0	3	0	0	6	POS
81	4	0	3	0	0	6	POS
94	4	0	3	0	0	6	POS
101	4	0	3	0	0	6	POS
112	4	1	2	0	1	6	POS
139	4	2	1	1	0	6	SOP
162	4	2	1	1	0	6	SOP
166	4	2	1	1	0	6	SOP
84	4	1	2	0	1	7	POS
98	4	1	2	0	1	7	POS
105	4	1	2	0	1	7	POS

Table 9.2: Brief description of 15 schemes, automatically generated by our tool, alternative to the circuit implemented by Rinaudo *et al.* [19].

abcd	Boolean output	Rinaudo's output	Our output
0000	0	0.03	0.0012
0001	0	0.12	0.0012
0010	0	0.03	0.0012
0100	1	1.00	1.0000
1000	0	0.03	0.0015
0011	0	0.22	0.0019
0101	1	1.11	1.0000
1001	0	0.32	0.0015
0110	1	1.01	1.0000
1010	0	0.03	0.0015
1100	0	0.03	0.0019
0111	1	1.28	1.0001
1011	1	1.20	1.0210
1101	0	0.18	0.0019
1110	0	0.02	0.0019
1111	1	1.02	1.0210

Table 9.3: Comparison between experimental data by Rinaudo *et al.* [19] and our computational results (the circuits are represented in Fig. 5 in the main text). Measurements refer to the luminous relative intensity of the fluorescent protein employed as an output. Our data have been properly rescaled to make the comparison possible. An estimation of the *relative* signal separation (σ_{rel}) can be inferred from these data sets. Our circuit solution appears to reach almost $\sigma_{rel} = 100\%$ (versus $\sigma_{rel} = 68\%$ in Rinaudo's circuit). However, this result is strictly dependent on our choice of default parameter values.

Solution	σ (M)	ρ (%)
**Promoter leakage 1%**		
$s1$	$2.66E-08$	6.27
$s2$	$2.02E-08$	6.98
$s3$	$2.15E-08$	12.07
$s4$	$3.86E-08$	3.62
$s25$	$5.25E-08$	17.10
$s26$	$3.03E-08$	5.29
$s27$	$2.74E-08$	11.38
$s28$	$4.85E-08$	4.41
**Promoter leakage 2%**		
$s1$	$1.95E-08$	11.42
$s2$	$9.61E-09$	17.02
$s3$	$1.43E-08$	23.61
$s4$	$1.54E-08$	6.60
$s25$	$4.27E-08$	32.83
$s26$	$2.87E-08$	11.38
$s27$	$1.81E-08$	21.74
$s28$	$5.21E-08$	8.50
**Promoter leakage 3%**		
$s1$	$1.48E-08$	18.12
$s2$	$4.03E-09$	38.07
$s3$	$8.24E-09$	38.69
$s4$	$3.40E-09$	20.32
$s25$	$3.43E-08$	46.15
$s26$	$2.70E-08$	17.95
$s27$	$1.24E-08$	37.21
$s28$	$4.41E-08$	27.44
**Promoter leakage 4%**		
$s1$	$1.15E-08$	26.02
$s2$	$1.77E-09$	63.44
$s3$	$4.43E-09$	53.61
$s4$	$5.43E-10$	58.58
$s25$	$2.76E-08$	56.78
$s26$	$2.52E-08$	24.61
$s27$	$7.93E-09$	58.35
$s28$	$1.10E-08$	82.38
**Promoter leakage 5%**		
$s1$	$9.12E-09$	34.71
$s2$	$8.83E-10$	80.94
$s3$	$2.48E-09$	64.77
$s4$	$1.43E-11$	98.07
$s25$	$2.24E-08$	65.10
$s26$	$2.33E-08$	31.13
$s27$	$3.13E-09$	84.24
$s28$	$-1.17E-09$	101.86

Table 9.4: Effect of promoter leakage; σ indicates the signal separation given by min1-max0; ρ the max0/min1 ratio.

Solution	σ (M)	ρ (%)
**RBS leakage 1%**		
$s1$	$2.58E-08$	6.45
$s2$	$2.18E-08$	7.47
$s3$	$5.75E-10$	92.88
$s4$	$5.24E-08$	5.08
$s25$	$-8.82E-09$	205.05
$s26$	$-1.45E-09$	223.00
$s27$	$-1.52E-09$	159.84
$s28$	$-2.40E-10$	120.22
**RBS leakage 2%**		
$s1$	$1.46E-08$	29.84
$s2$	$1.00E-08$	38.08
$s3$	$8.00E-09$	45.10
$s4$	$2.96E-08$	30.75
$s25$	$-2.19E-08$	299.84
$s26$	$-4.90E-09$	409.88
$s27$	$-1.18E-08$	545.83
$s28$	$-2.19E-09$	280.96
**RBS leakage 3%**		
$s1$	$-1.12E-09$	106.90
$s2$	$-5.63E-09$	148.11
$s3$	$-1.08E-08$	260.57
$s4$	$-1.67E-08$	153.95
$s25$	$-2.62E-08$	250.49
$s26$	$-7.98E-09$	392.80
$s27$	$-3.21E-08$	1132.21
$s28$	$-1.70E-08$	1407.99
**RBS leakage 4%**		
$s1$	$-4.51E-09$	134.49
$s2$	$-8.71E-09$	197.95
$s3$	$-1.01E-08$	368.15
$s4$	$-2.71E-08$	223.41
$s25$	$-2.68E-08$	214.19
$s26$	$-1.08E-08$	368.59
$s27$	$-3.94E-08$	1042.01
$s28$	$-5.78E-08$	4115.79
**RBS leakage 5%**		
$s1$	$-4.26E-09$	139.27
$s2$	$-8.05E-09$	214.29
$s3$	$-1.28E-08$	533.93
$s4$	$-2.96E-08$	285.83
$s25$	$-2.58E-08$	189.38
$s26$	$-1.33E-08$	346.21
$s27$	$-3.64E-08$	584.29
$s28$	$-6.77E-08$	4233.26

Table 9.5: Effect of RBS leakage; σ indicates the signal separation given by min1-max0; ρ the max0/min1 ratio.

Bibliography

[1] A. Arkin, J. Ross, and H. H. McAdams. Stochastic kinetic analysis of developmental pathway bifurcation in phage lambda-infected Escherichia coli cells. *Genetics*, 149(4):1633–1648, Aug 1998.

[2] Lacramioara Bintu, Nicolas E Buchler, Hernan G Garcia, Ulrich Gerland, Terence Hwa, Jan Kondev, Thomas Kuhlman, and Rob Phillips. Transcriptional regulation by the numbers: applications. *Curr Opin Genet Dev*, 15(2):125–135, Apr 2005.

[3] H. Bremer and P. P. Dennis. Modulation of Chemical Composition and Other Parameters of the Cell by Growth Rate. In F.C. Neidhardt, R. Curtiss III, J. L. Ingraham, E. C. C. Lin, K. Brooks Low, B. Magasanik, W. S. Reznikoff, M. Riley, M. Schaechter, and H. E. Umbarger, editors, *Escherichia coli and Salmonella typhimurium, Cellular and Molecular Microbiology*, pages 1553–1569. American Society for Microbiology, Whasington DC, 1996.

[4] R. A. Calogero, C. L. Pon, M. A. Canonaco, and C. O. Gualerzi. Selection of the mrna translation initiation region by Escherichia coli ribosomes. *Proc Natl Acad Sci U S A*, 85(17):6427–6431, Sep 1988.

[5] M. B. Elowitz and S. Leibler. A synthetic oscillatory network of transcriptional regulators. *Nature*, 403(6767):335–338, Jan 2000.

[6] T. Franch, M. Petersen, E. G. Wagner, J. P. Jacobsen, and K. Gerdes. Antisense RNA regulation in prokaryotes: rapid RNA/RNA interaction facilitated by a general U-turn loop structure. *J Mol Biol*, 294(5):1115–1125, Dec 1999.

[7] D. V. Freistroffer, M. Y. Pavlov, J. MacDougall, R. H. Buckingham, and M. Ehrenberg. Release factor RF3 in E.coli accelerates the dissociation of release factors RF1 and RF2 from the ribosome in a GTP-dependent manner. *EMBO J*, 16(13):4126–4133, Jul 1997.

[8] T. S. Gardner, C. R. Cantor, and J. J. Collins. Construction of a genetic toggle switch in Escherichia coli. *Nature*, 403(6767):339–342, Jan 2000.

[9] M. A. Gibson and J. Bruck. Efficient Exact Stochastic Simulation of Chemical Systems with Many Species and Many Channels. Caltech Parallel and Distributed Group technical report 026, Caltech, 1999.

[10] Stefan Hoops, Sven Sahle, Ralph Gauges, Christine Lee, Jrgen Pahle, Natalia Simus, Mudita Singhal, Liang Xu, Pedro Mendes, and Ursula Kummer. COPASI–a COmplex PAthway SImulator. *Bioinformatics*, 22(24):3067–3074, Dec 2006.

[11] Sara Hooshangi, Stephan Thiberge, and Ron Weiss. Ultrasensitivity and noise propagation in a synthetic transcriptional cascade. *Proc Natl Acad Sci U S A*, 102(10):3581–3586, Mar 2005.

[12] Farren J Isaacs, Daniel J Dwyer, Chunming Ding, Dmitri D Pervouchine, Charles R Cantor, and James J Collins. Engineered riboregulators enable post-transcriptional control of gene expression. *Nat Biotechnol*, 22(7):841–847, Jul 2004.

[13] M. Lanzer and H. Bujard. Promoters largely determine the efficiency of repressor action. *Proc Natl Acad Sci U S A*, 85(23):8973–8977, Dec 1988.

[14] B. Lewin. *genes VII.* Oxford University Press, New York, 2000.

[15] Maumita Mandal, Mark Lee, Jeffrey E Barrick, Zasha Weinberg, Gail Mitchell Emilsson, Walter L Ruzzo, and Ronald R Breaker. A glycine-dependent riboswitch that uses cooperative binding to control gene expression. *Science*, 306(5694):275–279, Oct 2004.

[16] M. A. Marchisio and J. Stelling. Computational design of synthetic gene circuits with composable parts. *Bioinformatics*, 24(17):1903–1910, Sep 2008.

[17] Eric Massé, Freddy E Escorcia, and Susan Gottesman. Coupled degradation of a small regulatory RNA and its mRNA targets in Escherichia coli. *Genes Dev*, 17(19):2374–2383, Oct 2003.

[18] Renate Rieder, Kathrin Lang, Dagmar Graber, and Ronald Micura. Ligand-induced folding of the adenosine deaminase a-riboswitch and implications on riboswitch translational control. *Chembiochem*, 8(8):896–902, May 2007.

[19] Keller Rinaudo, Leonidas Bleris, Rohan Maddamsetti, Sairam Subramanian, Ron Weiss, and Yaakov Benenson. A universal rnai-based logic evaluator that operates in mammalian cells. *Nat Biotechnol*, 25(7):795–801, Jul 2007.

[20] Henning Schmidt and Mats Jirstrand. Systems biology toolbox for matlab: a computational platform for research in systems biology. *Bioinformatics*, 22(4):514–515, Feb 2006.

[21] Rafael Silva-Rocha and Vctor de Lorenzo. Mining logic gates in prokaryotic transcriptional regulation networks. *FEBS Lett*, 582(8):1237–1244, Apr 2008.

[22] F Streichert and H Ulmer. Javaeva: A java based framework for evolutionary algorithms. Technical Report WSI-2005-06, Wilhelm-Schickard-Institut für Informatik (WSI), Centre for Bioinformatics Tübingen (ZBIT), Eberhard-Karls-University Tübingen, Germany., 2005.

[23] J. Tomsic, L. A. Vitali, T. Daviter, A. Savelsbergh, R. Spurio, P. Striebeck, W. Wintermeyer, M. V. Rodnina, and C. O. Gualerzi. Late events of translation initiation in bacteria: a kinetic analysis. *EMBO J*, 19(9):2127–2136, May 2000.

[24] Lisa M Tuttle, Howard Salis, Jonathan Tomshine, and Yiannis N Kaznessis. Model-driven designs of an oscillating gene network. *Biophys J*, 89(6):3873–3883, Dec 2005.

[25] J. Kenneth Wickiser, Ming T Cheah, Ronald R Breaker, and Donald M Crothers. The kinetics of ligand binding by an adenine-sensing riboswitch. *Biochemistry*, 44(40):13404–13414, Oct 2005.

[26] Wade C Winkler and Ronald R Breaker. Regulation of bacterial gene expression by riboswitches. *Annu Rev Microbiol*, 59:487–517, 2005.
